# Advanced analysis of nonlinear stability of two horizontal interfaces separating three-stratified non-Newtonian liquids

**DOI:** 10.1038/s41598-025-24182-6

**Published:** 2025-11-18

**Authors:** Galal M. Moatimid, Yasmeen M. Mohamed

**Affiliations:** Department of Mathematics, Faculty of Education, Ain Shams University, Roxy, Cairo, Egypt

**Keywords:** Nonlinear stability, Electrohydrodynamics, Casson liquid, Powell–Eyring liquid, Viscous potential flow, Non-perturbative approach

## Abstract

The nonlinear stability of two horizontal interfaces of three-layered stratified non-Newtonian fluids plays a pivotal role in advanced engineering applications. This phenomenon encompasses temperature management systems, microfluidic devices, and precise coating technologies. In an existing study, a multilayer system is considered wherein a central Casson liquid (CL) layer is bounded above and below by Powell–Eyring liquids (PELs). The impact of a uniform tangential electric field (EF) and surface tension is explored within a porous medium. To avoid the mathematical complexity, the viscous potential flow (VPF) is used to simplify the governing hydrodynamic formulations. The model involves Navier–Stokes and Maxwell equations under the quasi-static assumption. To obtain a nonlinear formulation, the linearized regulator equations are derived subject to appropriate nonlinear boundary conditions. The plan interfaces are presumed to propagate horizontally. To handle the nonlinear ordinary differential equations (ODEs) arising from the analysis, He’s frequency formula (HFF) is applied, transforming the problem into linear forms suitable for a non-perturbative approach (NPA). A non-dimensional analysis introduces key dimensionless collections, which help to characterize underlying fluid behavior and reduce system intricacy. A brief methodological summary of NPA is included to support reproducibility and clarity. The numerical calculations indicate that the stability can be evidently improved by the orientation of the tangential EF in relation to the horizontal wavenumber. PolarPlots are employed to imagine the influence of varying parameters, offering valuable insights into the mechanisms of the governing interfacial stability.

**Supplementary Information:**

The online version contains supplementary material available at 10.1038/s41598-025-24182-6.

## Introduction

Electrohydrodynamics (EHD) plays a vital role across various scientific and industrial applications. They affect hydrodynamics, droplet and bubble dynamics, atmospheric phenomena, and thunderstorm electrostatics^1^. Numerous studies have explored mechanisms driving EHD instabilities. For instance, the influence of an oblique magnetic field on Rayleigh–Taylor instability (RTI) in viscous, electrically conducting fluids revealed that the strength and the fluid layer’s thickness significantly affect stability^2^. Kelvin–Helmholtz instability (KHI) under mass and heat transfer constraints under oblique EFs was modelled mathematically^3^. Field-induced surface waves have also been observed at fluid interfaces under externally applied EFs^4^. The linear capillary instability of a cylindrical interface between two viscous dielectric fluids subjected to an axial EF was examined^5^. Additional studies addressed EHD instabilities in viscous liquids within cylindrical tubes subjected to perpendicular EFs^6^ and the linear surface wave behavior in leaky dielectric systems over finite fluid layers under varying EFs^7^. Experimental work using horizontal capacitors demonstrated distinct modes of EHD motion in low-conductivity fluids^8^. The methodology of the current work is entirely distinct from all prior research. The present study examines the weakly nonlinear stability of coupled interfaces using a novel technique referred to as NPA, as previously indicated in the Abstract.

The increasing technological relevance of non-Newtonian fluids stems from their greater ability to represent complex fluid behavior more accurately than Newtonian models. Viscoelastic fluids exhibit both viscous and elastic responses; they are critical in industries such as food processing, paper production, and petroleum recovery^9^. The stability of interfaces between such fluids is of particular interest due to its implications in multilayer flow systems across diverse applications. Several studies address interfacial stability in second-order and viscoelastic fluids, often under the influence of horizontal magnetic fields, employing non-Newtonian models such as Walters’ B fluid^10^. The development of more generalized models, particularly of polymeric and glass-forming fluids, was reported^11^. The Oldroyd-B fluid model, due to its wide applicability across geophysics, biomedicine, chemistry, and petroleum engineering, was extensively employed^12^. Energy transfer mechanisms reveal that traditional approaches may sometimes violate theoretical energy bounds. Further research has investigated the linear stability of electrified interfaces between coaxial Oldroyd-B fluids, incorporating effects of interfacial surfactants and surface charge distributions^13^. The present analysis precisely investigates complex rheological properties of non-Newtonian fluids, differentiating it from earlier research that mostly focused on Newtonian or mildly viscous flow models. This study distinguishes itself from prior studies by incorporating nonlinear constitutive relations pertinent to non-Newtonian fluids, which were omitted in earlier analyses predicated on simplistic Newtonian assumptions.

The concept of VPF in viscous liquids was first introduced by Stokes in 1851. His work focused on the impact of viscosity on the damping of small-amplitude waves at the liquid–gas interface. All relevant assertions from his study are referenced herein. Stokes’ issue was accurately addressed by employing linearized Navier–Stokes equations without the explicit proposition of VPF, as previously demonstrated^14–16^. Traditional potential inflow models, which presume perfect performance, are known to overestimate resonance impacts in water wave issues due to their cancellation of viscous dissipation. To address this, a modified potential influx model that incorporates viscous damping influences was proposed and validated^17^. A method of evaluating propeller-effective wakes in oblique inflow constraints was developed^18^. Furthermore, a hybrid model combining the boundary element method of wave-making resistance, empirical formulas of viscous resistance, and boundary layer theory was established to accurately predict the performance of water jet propulsion systems^19^. The nonlinear KHI of Rivlin–Ericksen viscoelastic electrified fluid–particle mixtures saturating porous media was explored^20^. The analysis considered the combined impacts of fluid elasticity, particle interactions, and porosity under an applied EF. The existing study examines VPF through the NPA, setting it apart from all earlier works.

Nonlinear oscillations play a pivotal role in understanding a wide range of complex phenomena across physics, electrical engineering, and modern manufacturing. Their solutions are often embedded in central physical principles and are closely linked to various natural and engineering processes. In recent years, iterative techniques such as the Homotopy perturbation method (HPM) have gained importance in producing approximate solutions of nonlinear problems with high accuracy, often approaching exact analytical results^21^. However, due to the inherent complexity of nonlinear systems, obtaining accurate or semi-analytical solutions of many nonlinear ODEs remains a significant challenge. Subsequent studies have addressed damped ODEs with higher-order nonlinearities using both computational and analytical methodologies^22^. The frequency–amplitude relationship in such systems can be explored via HFF, which was compared against its variants through residual analysis^23^. Various strategies of residual minimization were proposed to accurately determine the frequency of nonlinear oscillators, with outcomes closely tied to the reliability of HFF^24^. While the method yields satisfactory frequency predictions, there remains an opportunity for further modification^25^. Notably, HFF was successfully applied to an un-damped Duffing oscillator as well^26^. More recently, NPA has emerged as a powerful tool in both dynamical system analysis and hydrodynamic stability research^27–30^. Given the characteristic difficulties in analyzing similar nonlinear systems, the continued development and application of methods like NPA are essential. Accordingly, the present study adopts NPA as a suitable and robust framework for investigating nonlinear oscillatory behavior.

The nonlinear stability of two horizontal interfaces separating three stratified non-Newtonian fluids holds substantial experimental and practical significance across a range of scientific and engineering applications. This analysis is especially pertinent in microfluidic systems. These encompass lab-on-a-chip technologies utilized in biochemical tests, targeted medication administration, and diagnostics. In these systems, the regulation and stability of immiscible, stratified non-Newtonian fluids are essential in maintaining dependable performance during disturbances. In petroleum engineering, this research supports advanced oil recovery techniques where nanofluids are injected into layered reservoirs. Now, controlled interfacial instability can enhance mixing and mobilize trapped hydrocarbons. In thermal management technologies such as nanofluid-based heat exchangers, it is essential to maintain stable stratification. This stability aids in averting weakening in heat transfer efficiency due to interfacial disturbances. Furthermore, nonlinear stability analysis aids in the design of energy systems. It guarantees the structural integrity of stratified fluids under dynamic operational conditions. The theoretical insights gained directly support the development of efficient, stable, and adaptable fluid systems across industries. Experimentally, this investigation enables a deeper understanding of complex interfacial dynamics in systems dominated by non-Newtonian behavior. By examining nonlinear mechanisms governing stability under complex rheological conditions, this study aims to enhance prediction, control, and innovation in both theoretical and applied fluid dynamics. Accordingly, this work addresses the following key research queries:

1. What methods can be employed to derive the formulas of nonlinear stability of double interfaces?
2. What is the technique of the nonlinear algorithm in analyzing two degrees of freedom?
3. What is the current status of validation of the developed NPA?
4. What is the total number of non-dimensional physical quantities?
5. What are the prerequisites for the nonlinear stability of the double interfaces?
6. What is the impact of the non-dimensional physical numbers on the stability configuration?

The main objective of this work is to explore the nonlinear stability of two horizontal interfaces separating three stratified non-Newtonian fluid layers. The problem has been rarely explored compared to single-interface or Newtonian fluid cases. By employing a combined NPA and HFF, the study introduces a systematic algorithm that transforms nonlinear governing equations into manageable linear forms, providing deeper insights into the dynamics of multi-interface systems. Physically, the work captures the complex interplay between yield stress, shear thinning, and stress-limited viscosity in CL and PEL, thereby revealing essential conditions in maintaining or destabilizing stratification. The applications are broad and significant: in microfluidic technologies such as lab-on-a-chip platforms, controlled interfacial stability ensures reliable biochemical testing and targeted drug delivery; in nanofluid-based thermal management systems, sustaining stable interfaces prevents deterioration of heat transfer efficiency. Despite extensive studies on two-layer configurations and Newtonian or single-type of non-Newtonian fluids, the nonlinear stability of three-layer systems involving Casson and Powell–Eyring fluids under the influence of a tangential EF has not yet been systematically examined. This research gap provides the primary motivation of the present study. To clarify the manuscript’s organization, the structure is outlined as follows: A flowchart illustrating NPA is presented prior to § 2. It introduces the theoretical framework of the study. § 3 provides a detailed analysis of the nonlinear characteristics of the governing ODEs. § 4 focuses on the investigation of nonlinear stability. § 5 presents Tabulated and graphical validations, along with a comprehensive discussion of the results. § 6 provides a discussion of the outcomes. Lastly, § 7 summarizes the key findings and conclusions.

**Fig. 1:**
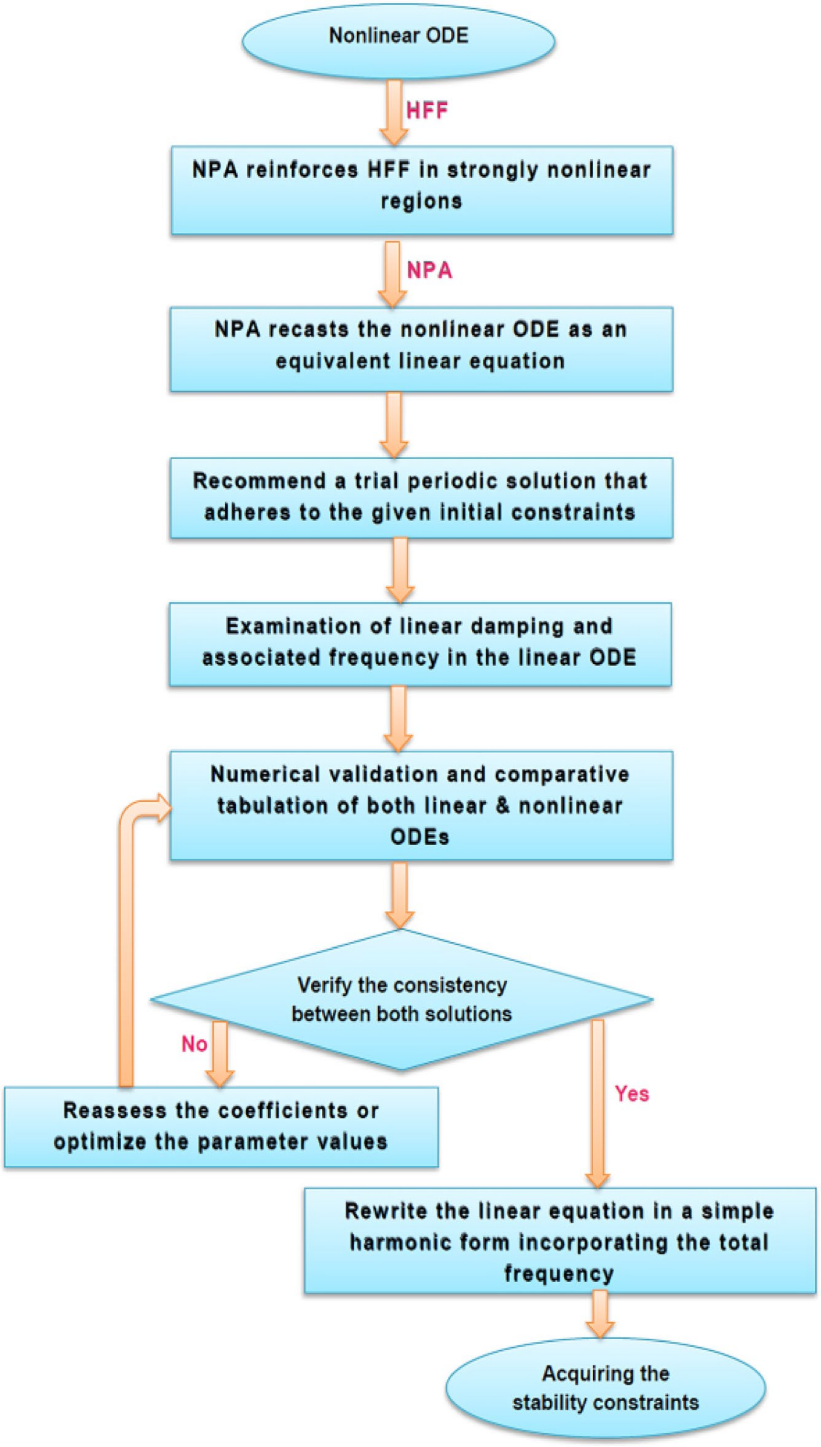
Schematic representation of the procedure, illustrating the integration of NPA with HFF.

To improve clarity and aid comprehension, the NPA procedure is presented in the following step-by-step flowchart. This visual representation highlights key stages of methodology, focusing on processes that streamline and enhance strategy of problem-solving strategy. Therefore, Fig. 1 elucidates this comprehensive flowchart, detailing the systematic approach employed to examine a nonlinear ODE through NPA combined with HFF. The procedure is designed to transform nonlinear ODEs into equivalent linear ones, simplifying the analysis. This transformation begins by proposing a trial solution, which is then thoroughly evaluated through numerical simulations and summarized employing Tabulated data. The subsequent points outline particular characteristics of this methodology:

1. NPA is fundamentally different from any perturbation technique, including multiple time scales or HPM. The NPA serves exclusively as an alternate tactic.
2. The concept is objective and originates from HFF. Certainly, ancient Chinese mathematicians were the forerunners of this discovery.
3. The objective of this concept is to achieve a linear ODE that corresponds to the nonlinear one.
4. The numerical compatibility of ODEs ensures their consistency.
5. The linear ODE includes all parameters that are found in the nonlinear ODE.
6. NPA did not highlight that it had analytically resolved the nonlinear ODE.
7. NPA employs a distinctive methodology in addressing restoring forces, diverging from the conventional perturbation approach; it is not classified as a perturbation technique.
8. Taylor expansion is employed to facilitate the calculation of restoring forces in all perturbation methods, including the innovative multiple time scale technique. The NPA disregarded this risk.
9. The subsequent research utilized NPA to analyze the coupled systems, recognizing their importance.

## Theoretical framework

For more clarification, this section will be divided as follows:

### Mathematical description

**Fig. 2:**
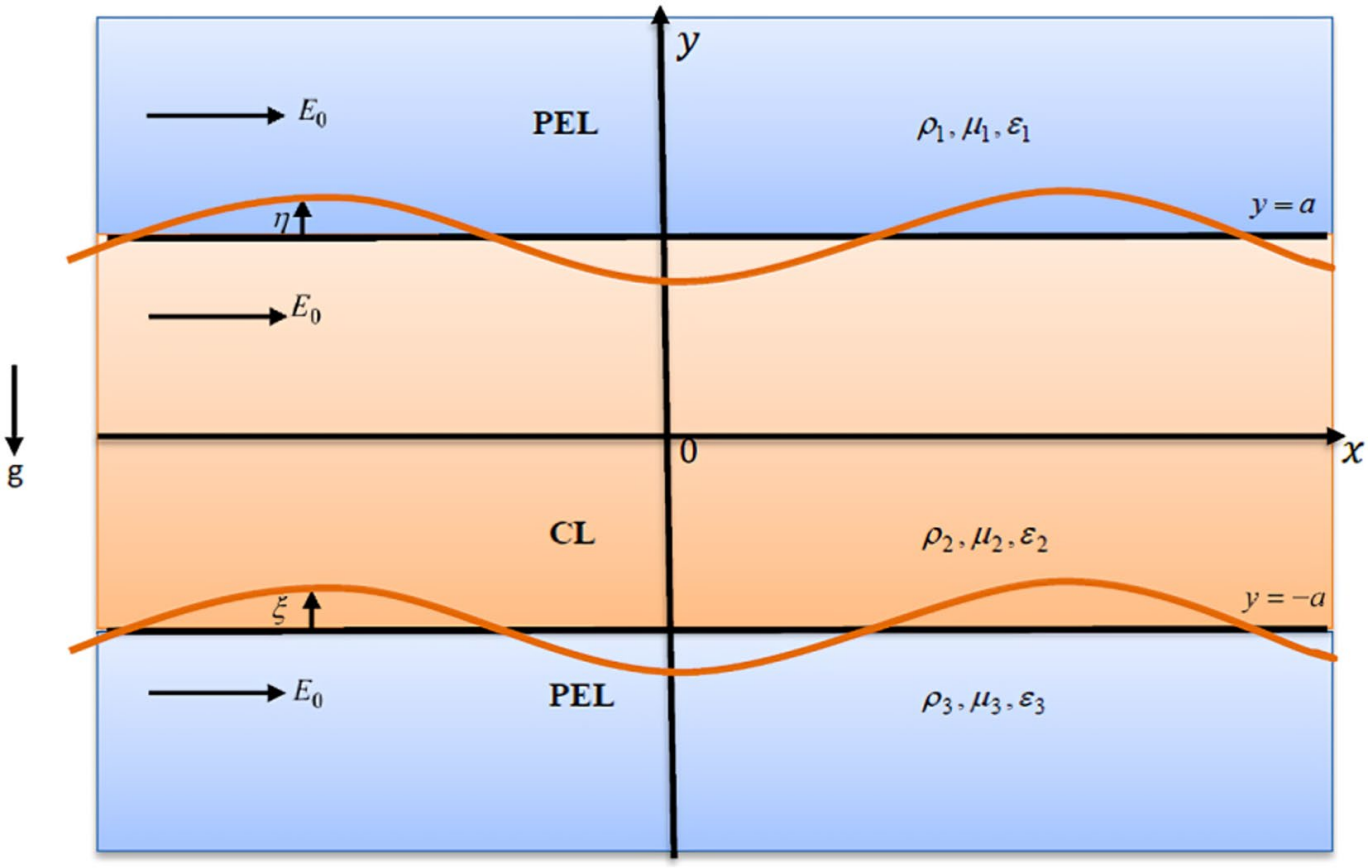
Schematic representation of the theoretical model.

The nonlinear stability of double interfaces separating three immiscible liquids, PEL, CL, and PEL, is formulated to examine the evolution and stability of interfacial perturbations. The perturbations in each liquid layer are governed by nonlinear equations of movement, which include surface tension influences. In the steady-state (equilibrium) configuration, the system features two distinct, flat interfaces, which are located at $y = a$ and $y = - a$ and separating three liquid non-Newtonian layers. As demonstrated in Fig. 2, the upper and lower layers consist of a shear-thinning liquid modeled by PEL, characterized by a viscosity that diminishes with escalating shear rate. The middle layer comprises a yield-stress liquid represented by the CL model. This liquid behaves as a solid until the applied stress surpasses a critical threshold, after which it flows. Such characteristics are common in materials like pastes and observed in biological and industrial liquids such as blood and paints. A uniform tangential EF is applied horizontally, introducing an EHD impact that further modifies the interface dynamics and stability conduct of the system. The gravity $\underline{g} = - g\underline{e}_{y}$ acts vertically downward in the direction of the *y*-axis. The three liquids are confined within a permeable medium, where the flow performance is governed by Darcy’s law. This empirical law relates fluid velocity to pressure gradient across a medium, accounting for the permeability of the medium and viscosity of the liquid. Darcy’s law governs the transition of liquids through porous structures, linking micro-scale fluid–solid interactions to macroscale inflow dynamics. Accordingly, it plays a vital role in bridging fluid dynamics with underlying physiological and industrial processes. This analysis is conducted within a Cartesian coordinate framework $(x,\,y)$. In this setting, the subscripts $1,\,2,\,{\text{and}}\,3$ are applied to denote the constraints corresponding to upper, central, and lower liquids, respectively, which are adjacent to the fixed boundary surfaces.

### Elucidation of physical mechanism

The study of interface stability examines systems in which two liquid layers are separated by distinct boundaries, each defined by different physical properties such as density or velocity. Gaining insight into the stability of these interfaces is crucial for numerous engineering and biological applications. Prominent examples include enhanced oil recovery, blood perfusion in tissues, targeted drug delivery systems, soft robotics, and the manufacturing of paints and coatings. Consequently, the concept of interfacial stability plays a vital role across a wide spectrum of scientific and technological fields, including the following applications:

#### Medical applications

**Fig. 3:**
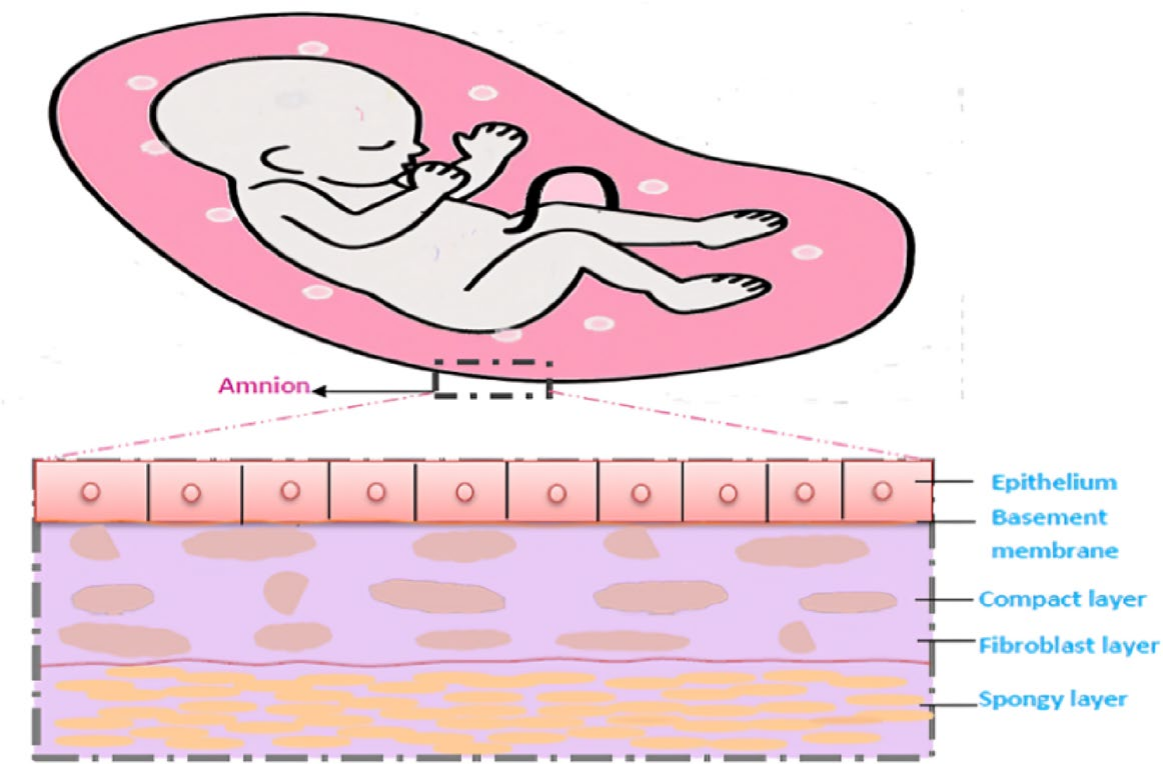
Representation of a real-world application featuring three distinct liquid layers.

Figure 3 shows a depiction of a developing human embryo within the uterus, enclosed by the chorioamniotic membrane. This membrane is composed of two primary layers: the outer chorion, which interfaces with maternal cells, and the inner amniotic membrane, which directly surrounds the embryo. A gelatinous matrix separates these two layers. The amniotic membrane itself is structured into three sub-layers: the epithelium, the basement membrane, and the stroma. The stroma is further differentiated into compact, fibroblast, and sponge layers^31^.

#### Geological applications

**Fig. 4:**
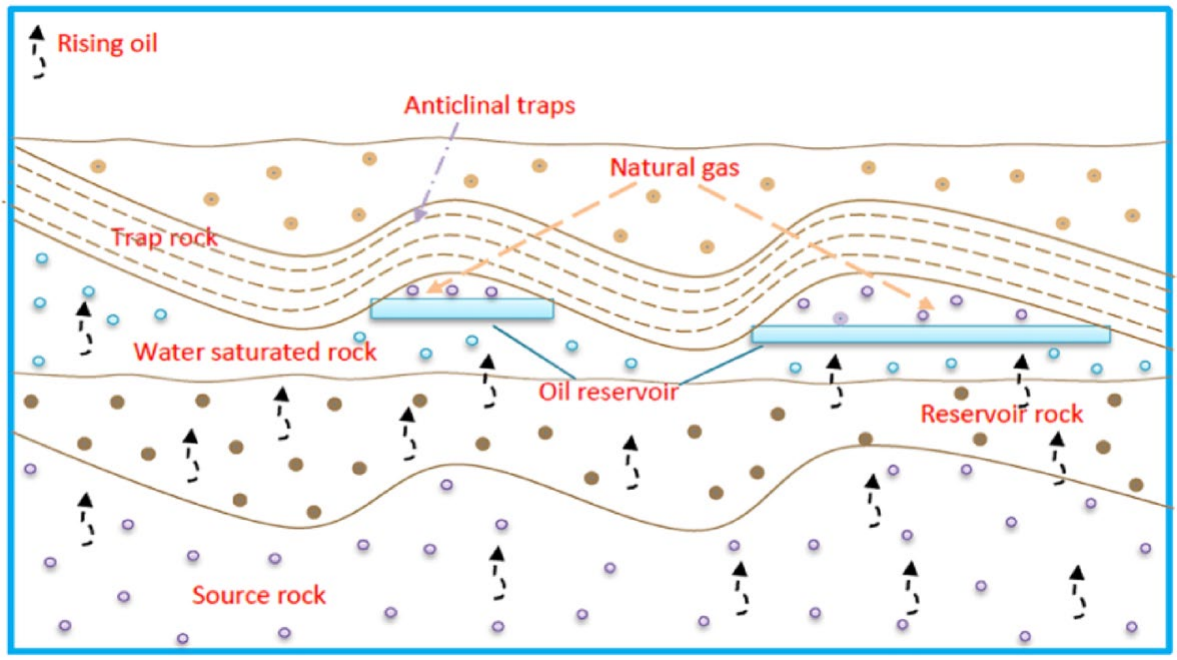
Geological mechanisms underlying oil and gas accumulation.

The investigation of double interfaces holds particular importance in groundwater management and geothermal energy systems. In groundwater contexts, these interfaces separate contaminants, clean water, and geological fluids, thereby influencing the migration of pollutants. Understanding these interactions supports the prediction of contaminant behavior and the development of remediation strategies such as filtration or chemical treatment. In geothermal systems, interfaces arise between hot geothermal fluids and cooler extraction fluids, directly affecting heat transfer and fluid inflow. Insights into these mechanisms contribute to optimizing energy recovery and maintaining reservoir stability. Furthermore, Fig. 4 exhibits the natural accumulation of oil and gas through the coordinated interaction of geological elements, source rocks, reservoirs, seals, and traps, governing hydrocarbon migration, storage, and entrapment^32^.

### Mathematical modelling framework

The displaced boundaries can be expressed as^27,29^:

1a $$y = a + \eta (x;t),$$

and

1b $$y = - a + \xi (x;t).$$

Any function subjected to perturbation can be formulated as^27,29^:

2 $$M(x,\;y;t) = \tilde{M}(y;t){\text{Exp}}(ikx).$$

The liquid’s momentum formula can be given as^27,28^:

3 $$\rho \left( {\frac{\partial }{\partial t} + (\underline{V} .\nabla )} \right)\underline{V} = - \nabla P - \rho g\underline{e}_{y} - \frac{{\mu_{j} }}{\alpha }\underline{V}_{j} ,$$

where the third term on the right-hand side, $- \frac{{\mu_{j} }}{\alpha }\underline{V}$, represents the Darcy resistance associated with the porous structure. In the present formulation, the EHD force does not appear as a bulk body force but rather enters through the modified interfacial BCs via Maxwell stresses. The pressure gradient is represented by $- \nabla P$, and various physical quantities are previously defined in the Nomenclature Table.

### Theoretical limitations of the issue

The limitations of both the VPF model and NPA can be clarified in more precise terms as follows:

1. Limitations of the VPF model

The VPF framework suffers from several fundamental drawbacks that arise from its central assumption of an inviscid flow field, which restricts its validity. In this model,

- Viscosity is only incorporated indirectly through normal stresses in the dynamic BC; meanwhile, its influence on the bulk fluid motion and tangential stresses is completely neglected.
- This simplification prevents VPF from accurately representing critical viscous flow features such as:
  - Boundary-layer development,
  - Flow separation,
  - Vortex shedding.
- The model’s accuracy deteriorates significantly at:
  1. High Reynolds numbers,
  2. Strongly rotating flows,
  3. Turbulent regimes,
  where vorticity generation and viscous dissipation are dominant.
- VPF fails to predict long-term energy dissipation and complex near-wall structures.

Consequently, it is only suitable for cases where viscosity exerts a minimal influence on the overall flow field.

2. Limitations of NPA

While NPA provides a sophisticated tool for analyzing nonlinear systems, its scope is also constrained by several factors:

1. Applicability: The method is specifically tailored for weakly nonlinear oscillators that can be described by a second-order ODE. It cannot be readily extended to strongly nonlinear systems or to higher-order dynamical models.
2. ICs: The technique inherently assumes that ICs remain fixed throughout the analysis, limiting flexibility in exploring broader ranges of dynamical responses.
3. Amplitude restriction: For the method to yield accurate and convergent results, the initial amplitude of oscillations must be less than unity. When this condition is not satisfied, the precision of the approach is compromised.

Accordingly, presupposing an irrotational influx and employing VPF, a velocity potential can be defined as $\varphi_{j} = \varphi_{j} (x,y;t)$ which can follows Laplace’s formula as:

4 $$\underline{{\text{V}}}_{j} = \nabla \varphi_{j} ,\nabla .\underline{{\text{V}}} = 0 \Rightarrow \nabla^{2} \varphi_{j} = 0,\;a + \eta < y < - a + \xi .$$

Subsequently, one gets:

5a $$\varphi_{1} (x,y;t) = A_{1} \,(t)\,e^{{k\left( {ix - y} \right)}} ,$$

5b $$\varphi_{2} (x,y;t) = A_{2} \,(t)\,e^{{k\left( {ix + y} \right)}} + A_{3} (t)\,\,e^{{k\left( {ix - y} \right)}} ,$$

and

5c $$\varphi_{3} (x,y;t) = A_{4} \,(t)\,e^{{k\left( {ix + y} \right)}} ,$$

where the constants $A_{1} (t) - \,A_{4} (t)$ are provided in the Appendix.

Employing VPF, $P_{j}$ can be given as:

6 $$P_{j} = - \rho_{j} \varphi_{jt} - \rho_{j} g\,y - \frac{{\mu_{j} }}{\alpha }\varphi_{j} .$$

Under the framework of EHD analysis, it is widely recognized that the quasi-static approximation is applicable, whereby the impacts of magnetic forces are deemed negligible. Accordingly, EF must be an irrotational, allowing the definition of electric scalar potentials $\psi_{j} (X,\,y,;\,t)\,$. Within liquid sheets $\underline{E} = E_{0} \underline{e}_{x} - \nabla \psi_{j}$, EF behaves accordingly. At the initial state, the free charge density within both liquids is presupposed to be zero, and this constraint remains constant over time. Based on Gauss’s law, the electric potentials are required to satisfy Laplace’s formula, as referenced in^27–30^.

7 $$\nabla^{2} \psi_{j} = 0,\,\,a + \eta < y < - a + \xi .$$

Thereby, one gets:

8a $$\psi_{1} (x,y;t) = B_{1} \,(t)\,e^{{k\left( {ix - y} \right)}} ,$$

8b $$\psi_{2} (x,y;t) = B_{2} \,(t)\,e^{{k\left( {ix + y} \right)}} + B_{3} (t)\,\,e^{{k\left( {ix - y} \right)}} ,$$

and

8c $$\psi_{1} (x,y;t) = B_{4} \,(t)\,e^{{k\left( {ix + y} \right)}} ,$$

where $B_{1} (t) - \,B_{4} (t)$ are moved to the Appendix.

The equations of $S_{a},$ and $S_{ - a}$ are expressed as follows:

9a $$S_{a} (x,\,\,y,;\,t) = y - a - \eta (x;\,t) = 0,$$

and

9b $$S_{ - a} (x,\,\,y,;\,t) = y + a - \xi (x;\,t) = 0.$$

The equations of $\underline{n}_{a}$ and $\underline{n}_{{\left( { - a} \right)}}$ can be computed by:

10a $$\underline{n}_{a} = \frac{{\nabla S_{a} }}{{\left| {\nabla S_{a} } \right|}} = \left( { - \eta_{x} ,\,1} \right)\left( {1 + \left( {\eta_{x} } \right)^{2} } \right)^{ - 1/2} ,$$

and

10b $$\underline{n}_{ - a} = \frac{{\nabla S_{ - a} }}{{\left| {\nabla S_{ - a} } \right|}} = \left( { - \xi_{x} ,\,1} \right)\left( {1 + \left( {\xi_{x} } \right)^{2} } \right)^{ - 1/2} .$$

The potentials $\phi_{j}$, and $\psi_{j}$ can be evaluated by employing the corresponding BCs^28–30^.

1. The kinematic constraints:
  11a $$\frac{{DS_{a} }}{Dt} = \frac{\partial \eta }{{\partial t}} + \frac{{\partial \phi_{1} }}{\partial y} - \left( {\frac{{\partial \phi_{1} }}{\partial x}} \right)\frac{\partial \eta }{{\partial x}} = 0,$$
  11b $$\frac{{DS_{a} }}{Dt} = \frac{\partial \eta }{{\partial t}} + \frac{{\partial \phi_{2} }}{\partial y} - \left( {\frac{{\partial \phi_{2} }}{\partial x}} \right)\frac{\partial \eta }{{\partial x}} = 0,$$
  11c $$\frac{{DS_{ - a} }}{Dt} = \frac{\partial \xi }{{\partial t}} + \frac{{\partial \phi_{2} }}{\partial y} - \left( {\frac{{\partial \phi_{2} }}{\partial x}} \right)\frac{\partial \xi }{{\partial x}} = 0,$$
  and
  11d $$\frac{{DS_{ - a} }}{Dt} = \frac{\partial \xi }{{\partial t}} + \frac{{\partial \phi_{3} }}{\partial y} - \left( {\frac{{\partial \phi_{3} }}{\partial x}} \right)\frac{\partial \xi }{{\partial x}} = 0.$$
2. Continuity of tangential EF across interfaces must be kept, which can be mathematically formulated as:
  12a $$\left. {\underline{E}_{oj} } \right|_{y = a + \eta } = \underline{n}_{a} \wedge \left\| {\underline{E}_{0} } \right\| = \underline{0} ,$$
  and
  12b $$\left. {\underline{E}_{oj} } \right|_{y = - a + \xi } = \underline{n}_{{\left( { - a} \right)}} \wedge \left\| {\underline{E}_{0} } \right\| = \underline{0} .$$
3. Continuity of normal EF across interfaces is required and can be expressed mathematically as:
  13a $$\left. {\underline{E}_{oj} } \right|_{y = a + \eta } = \underline{n} \,_{a} .\,\left\| {\varepsilon \underline{E}_{0} } \right\| = 0,$$
  and
  13b $$\left. {\underline{E}_{oj} } \right|_{y = - a + \xi } = \underline{n}_{{\left( { - a} \right)}} \cdot \left\| {\varepsilon \underline{E}_{0} } \right\| = 0.$$

The following section highlights the normal component of the stress tensor employed to evaluate the stability characteristics of the structure.

### Nonlinear BCs

For dynamic constraints, normal stresses must remain continuous across the disturbed boundaries, which are mathematically formulated as follows^28,30^:

14a $$\underline{n}_{a} .\left( {\underline{G}_{a} } \right) = T_{s} \nabla .\underline{n}_{a} ,\;{\text{at}}\;y = a + \eta ,$$

and

14b $$\underline{n}_{ - a} .\left( {\underline{G}_{ - a} } \right) = T_{s} \nabla .\underline{n}_{ - a} ,\;{\text{at}}\;y = - a + \xi ,$$

where $\underline{G}_{j}$ can be given as follows:

15 $$\underline{G}_{j} = \left( {\begin{array}{*{20}c} {\sigma_{xx}^{j} } & {\sigma_{xyj} } \\ {\sigma_{yx}^{j} } & {\sigma_{yyj} } \\ \end{array} } \right)\left( {\begin{array}{*{20}c} {n_{xj} } \\ {n_{yj} } \\ \end{array} } \right).$$

The expressions of $\sigma_{ij}^{total}$, $\sigma_{ij}^{hydro}$, and $\sigma_{ij}^{electro}$ can be given as follows^28,30^:

16a $$\sigma_{ij}^{total} = \sigma_{ij}^{hydro} + \sigma_{ij}^{electro} ,$$

16b $$\sigma_{ij}^{hydro} = - P_{j} \delta_{ij} + S_{ij} ,$$

and

17 $$\sigma_{ij}^{electro} = \varepsilon_{j} E_{0i} E_{0j} - \frac{1}{2}\varepsilon_{j} E_{0}^{2} \delta_{ij} .$$

This study explores three types of immiscible liquids, which can be described as follows:

### PEL in first and third layer

The PEL model reflects a category of non-Newtonian fluids that demonstrate both viscous and elastic characteristics, effectively capturing the complex mechanical behavior of many real-life matters. From a physical view, the model elucidates the dual performance of materials: under low shear stress, the substance resists inflow and acts like a solid; meanwhile, escalating stress induces a gradual shift toward fluid-like behavior. This stress-dependent response, commonly referred to as shear-thinning or pseudo-plasticity, is marked by a decrease in apparent viscosity with rising shear rate. Embedded in Eyring’s inflow theory, the PEL model accounts for the progressive yielding seen in complex fluids, unlike traditional Newtonian models, which presume a fixed viscosity. Its flexible structure makes it especially effective in modeling materials that transition smoothly from a solid-like to a liquid-like state, rather than exhibiting a distinct yield point.

To strengthen the theoretical framework, the constitutive equation for the PEL model may be formulated as^33–36^:

18 $$\begin{aligned} & \underline{S}_{PEL} = \mu_{i} \left( {\nabla \underline{V} + \left( {\nabla \underline{V} } \right)^{T} } \right) + \frac{1}{\gamma }\sinh^{ - 1} \left( {\frac{1}{\varsigma }\left( {\nabla \underline{V} + \left( {\nabla \underline{V} } \right)^{T} } \right)} \right),i=1,3, \\ & \quad \;\;\left| {\frac{1}{\varsigma }\left( {\nabla \underline{V} + \left( {\nabla \underline{V} } \right)^{T} } \right)} \right| < < 1, \\ \end{aligned}, $$

where $\underline{S}_{PEL}$ is the stress deviator, $\varsigma ,\,\gamma$ are defined as the material constants of the PEL model,

#### Specific cases of the PEL model

The special situations of the PEL model are given as:

1. At very high shear rates, the liquid conduct may approximate that of a Newtonian liquid with lower viscosity.
2. At low shear rates, the liquid may appear nearly solid.

These limiting performances provide useful approximations for tailoring the model to specific materials and constraints.

In terms of practical applications, the PEL model is highly relevant in describing complex biological and industrial fluids. It effectively captures the rheological behavior of blood, where the combination of plasma and suspended cells gives rise to distinct non-Newtonian characteristics. The model is also suitable for polymer melts and solutions, which deform under applied stress due to molecular entanglements. In biomedical contexts, it aids in simulating the mechanical response of soft tissues, where both viscous and elastic properties are critical to physiological function. Moreover, the PEL framework is valuable in various industrial processes, such as inkjet printing, food processing, and lubrication, where precise control of fluid flow is essential.

### CL in middle-layer

The CL model represents a category of non-Newtonian fluids distinguished by the presence of a yield stress, a critical threshold below which the material resists flow and behaves like a solid. Flow initiates only when applied shear stress surpasses this yield point, at which stage the material transitions into a viscous, flowing state. This behavior is particularly relevant to materials with internal microstructures, such as particle suspensions or cellular matrices, which withstand deformation until an adequate force is applied.

Mathematically, CL describes a nonlinear relationship between shear stress and shear rate. Once the yield stress is exceeded, shear stress becomes proportional to the square root of the shear rate. This framework effectively captures both initial rigidity and subsequent viscous response, making it a robust model for complex fluids that remain stationary under low stresses but flow readily once activated.

To further clarify the rheological framework, the constitutive equation for CL is given explicitly as^37–40^:

19 $$\underline{S}_{CL} = \left( {\begin{array}{*{20}l} {2\left( {\mu_{2} + \frac{{p_{y} }}{{\sqrt {2\pi } }}} \right)e,} \hfill & {\pi > \pi_{c} ,} \hfill \\ {2\left( {\mu_{2} + \frac{{p_{y} }}{{\sqrt {2\pi } }}} \right)e,} \hfill & {\pi < \pi_{c} ,} \hfill \\ \end{array} } \right.$$

where $\pi = e \cdot e = e_{ij} \,\,e_{ij}$ is the product of the component of deformation rate, $\pi_{c}$ is a critical value of this product based on a non-Newtonian model, $\mu_{2}$ is the plastic dynamic viscosity of the non-Newtonian fluid, and $p_{y}$ is the yield stress of the fluid.

#### Specific cases of the CL

The special situations of CL can be outlined as:

1. At very high shear rates, CL approximates Newtonian performance, as yield stress diminishes and fluid influxes more freely.
2. When liquid demonstrates a consistent increase in shear stress with shear rate after exceeding yield stress, the model approaches Bingham plastic performance.
3. CL is well-suited for fluids that exhibit yield stress, offering a realistic and flexible representation of such materials.

From a practical standpoint, CL is widely employed in both biological and industrial contexts. In hematology, it has been extensively used to simulate the rheological behavior of blood, where yield stress arises due to red blood cell aggregation, particularly under low shear conditions (e.g., in capillaries or at rest). This makes the model highly valuable in cardiovascular simulations and the design of medical devices such as blood pumps. In industry, CL is equally effective in describing pastes, emulsions, and suspensions, including chocolate, printing inks, cosmetics, and toothpaste, where material must resist flow when undisturbed but flow easily when processed or applied.

## Nonlinear characteristic equations

For the sake of simplicity, in the current case, temporal instability is explored by reducing PDEs to ODEs by applying perturbations on $\eta ,\,\xi$ as^28,29^:

20a $$\eta (x;t) = \eta (t)\,e^{ikx} ,$$

and

20b $$\xi (x;t) = \xi (t)\,e^{ikx} .$$

To examine nonlinear dynamics, analysis is conducted within a wave-attached reference system, where deformable interfaces are tackled purely as time-dependent. Presuming small surface disturbances, a binomial expansion is applied to simplify the nonlinear terms. While the algebra involved is extensive, the procedure follows a clear and logical structure. This approach leads to the derivation of nonlinear equations in terms of $\eta$ and $\xi$ and their time derivatives, accurate up to third-order terms. Consequently, one finds:

21 $$\begin{aligned} & \ddot{\eta } + r_{1} \ddot{\xi } + r_{2} \dot{\eta } + r_{3} \dot{\xi } + r_{4} \eta + r_{5} \xi + r_{6} \eta \ddot{\eta } + r_{7} \xi \ddot{\xi } + r_{8} \eta \ddot{\xi } \\ & + r_{9} \xi \ddot{\eta } + r_{10} \xi \dot{\eta } + r_{11} \eta \dot{\xi } + r_{12} \eta \dot{\eta } + r_{13} \xi \dot{\xi } + r_{14} \eta^{2} + r_{15} \xi^{2} \\ & + r_{16} \dot{\eta }\dot{\xi } + r_{17} \eta^{3} + r_{18} \xi^{3} + r_{19} \eta^{2} \ddot{\eta } + r_{20} \xi^{2} \ddot{\xi } + r_{21} \xi^{2} \ddot{\eta } \\ & + r_{22} \eta^{2} \ddot{\xi } + r_{23} \eta^{2} \dot{\eta } + r_{24} \xi^{2} \dot{\xi } + r_{25} \xi^{2} \dot{\eta } + r_{26} \eta^{2} \dot{\xi } = 0, \\ \end{aligned}$$

and

22 $$\begin{aligned} & \ddot{\xi } + s_{1} \ddot{\eta } + s_{2} \dot{\xi } + s_{3} \dot{\eta } + s_{4} \xi + s_{5} \xi \ddot{\xi } + s_{6} \eta \ddot{\eta } + s_{7} \eta \ddot{\xi } \\ & + s_{8} \xi \ddot{\eta } + s_{9} \eta \dot{\xi } + s_{10} \xi \dot{\eta } + s_{11} \xi \dot{\xi } + s_{12} \eta \dot{\eta } + s_{13} \xi^{2} + s_{14} \eta^{2} \\ & + s_{15} \dot{\eta }\dot{\xi } + s_{16} \xi^{3} + s_{17} \eta^{3} + s_{18} \xi^{2} \ddot{\xi } + s_{19} \eta^{2} \ddot{\eta } + s_{20} \eta^{2} \ddot{\xi } \\ & + s_{21} \xi^{2} \ddot{\eta } + s_{22} \xi^{2} \dot{\xi } + s_{23} \eta^{2} \dot{\eta } + s_{24} \eta^{2} \dot{\xi } + s_{25} \xi^{2} \dot{\eta } = 0, \\ \end{aligned}$$

where dashes reflect time derivatives. Further, coefficients $r_{1} \to r_{26}$ and $s_{1} \to s_{25}$ are moved to the Appendix.

For analytical clarity and to better capture governing physical effects, primary variables are expressed in dimensionless form. This transformation is carried out using a representative characteristic length, denoted as $l$. Employing this scale, the following non-dimensional factors are outlined to facilitate the interpretation of findings.

$$a^{*} = a/l$$, $$- a^{*} = \left( { - a} \right)/l$$, $$\Lambda = 1 + \beta_{c}^{ - 1}$$, $$k^{*} = k/l$$, $$\eta^{*} = \eta /l$$, $$\xi^{*} = \xi /l$$, $$E_{0}^{*2} = l\,\varepsilon_{2} \,E_{0}^{2} /T$$, $$\rho_{j}^{*} = \rho_{j} /\rho_{2}$$, $$t^{*} = \sqrt {T_{s} /\rho_{2} \,l^{3} } t,$$$$\varepsilon_{j}^{*} = \varepsilon_{j} /\varepsilon_{2}$$, and $$\mu_{j}^{*} = \mu_{j} /\mu_{2}$$, where the symbol asterisk is excluded for brevity.

Physically, expressing variables in dimensionless form eliminates unit dependence and highlights the balance of dominant physical mechanisms. This makes it possible to generalize results across systems of different scales and fluids, allowing predictions to be transferred from theory to experiments and applications.

Additionally, the non-dimensional physical parameters may be listed as:

- The Darcy number $D_{n} = \alpha /l^{2}$ represents the relative ease with which a fluid can flow through a porous medium compared to the viscous resistance of the fluid. A high Darcy number indicates high permeability; meanwhile, a low value indicates that the porous medium strongly restricts fluid motion.
- Ohnesorge number $Oh = \mu_{2} /\sqrt {\rho_{2} \,T_{s} \,l}$ compares viscous forces to the combined effects of inertial and surface tension forces. It is particularly important in understanding droplet formation, breakup, and interfacial stability, since a high value means viscous damping dominates the system’s dynamics.
- Bond number $B_{d} = \rho_{2} \,g\,\,l^{2} /T_{s}$ compares the influence of gravitational forces to the effect of surface tension forces acting on a fluid interface. A large Bond number means gravity dominates, leading to deformation or destabilization of the interface; meanwhile, a small Bond number means surface tension dominates, helping to maintain a stable and curved interface.
- EPL parameter $P_{e} = 1/\varsigma \gamma \mu_{2}$ characterizes how the viscosity of a non-Newtonian fluid becomes limited under stress. It reflects the degree of shear-thinning behavior and helps describe how a fluid’s resistance to flow decreases as stress increases.
- CL factor $\beta_{c} = p_{y}^{ - 1} \mu_{2} \sqrt {2\pi_{c} }$ used to describe the role of yield stress in the flow behavior of non-Newtonian fluids. It distinguishes between regions where fluid behaves like a solid, resisting motion until a threshold stress is exceeded, and regions where fluid flows once that yield stress is overcome.

## Nonlinear stability analysis

This section focuses on extending the previously established NPA to address nonlinear ODEs, as discussed in earlier studies^27,30^. Our analysis centers on Eqs. (21) and (22), which form a coupled nonlinear system. Due to the inherent complexity of these equations, we simplify the procedure by introducing specific substitution relationships. In particular, in Eq. (21), we replace the relevant terms with their corresponding expressions, and similarly, in Eq. (22), we substitute the necessary terms $$\eta = A\cos \varpi_{1} t,$$$$\xi = B\cos \varpi_{1} t$$ in Eq. (21) and $$\eta = A\cos \varpi_{2} t$$, $$\xi = B\cos \varpi_{2} t$$ in Eq. (22). This approach is implemented under the ICs, which will be defined as follows:

23 $$\eta (0) = A,\,\xi (0) = B,\,\eta^{\prime } (0) = 0\;and\;\xi^{\prime } (0) = 0.$$

These steps simplify the process and make it easier to tackle the equations. Subsequently, one finds:

24a $$\eta^{\prime \prime } + N_{1} (\eta ,\,\eta^{\prime } ,\xi ,\xi^{\prime } ) + N_{2} (\eta ,\,\eta^{\prime } ,\eta^{\prime \prime } ,\xi ,\,\,\xi^{\prime } ,\xi^{\prime \prime } ) = 0,$$

and

24b $$\xi^{\prime \prime } + D_{1} (\xi ,\xi^{\prime } ,\eta ,\eta^{\prime } ) + D_{2} (\xi ,\,\xi^{\prime } ,\xi^{\prime \prime } ,\eta ,\,\eta^{\prime } ,\eta^{\prime \prime } ) = 0,$$

where

25 $$\begin{aligned} & N_{1} (\eta ,\,\eta^{\prime } ,\eta^{\prime \prime } ,\xi ,\,\,\xi^{\prime } ,\xi^{\prime \prime } ) = r_{2} \eta^{\prime } + r_{3} \xi^{\prime } + r_{10} \xi \eta^{\prime } + r_{11} \eta \xi^{\prime } + r_{12} \eta \eta^{\prime }\\ & \quad + r_{13} \xi \xi^{\prime } + r_{16} \eta^{\prime } \xi^{\prime }+ r_{23} \eta^{2} \eta^{\prime } + r_{24} \xi^{2} \xi^{\prime } + r_{25} \xi^{2} \eta^{\prime }  + r_{26} \eta^{2} \xi^{\prime }  , \\ \end{aligned}$$

26 $$\begin{aligned} & N_{2} (\eta ,\,\eta^{\prime } ,\eta^{\prime \prime } ,\xi ,\,\,\xi^{\prime } ,\xi^{\prime \prime } ) = r_{1} \xi^{\prime \prime } + r_{4} \eta + r_{5} \xi+ r_{6} \eta \eta^{\prime \prime } + r_{7} \xi \xi^{\prime \prime } + r_{8} \eta \xi^{\prime \prime } + r_{9} \xi \eta^{\prime \prime } + r_{14} \eta^{2} \\ & \quad + r_{15} \xi^{2}  + r_{17} \eta^{3} + r_{18} \xi^{3} + r_{19} \eta^{2} \eta^{\prime \prime } + r_{20} \xi^{2} \xi^{\prime \prime } + r_{21} \xi^{2} \eta^{\prime \prime } + r_{22} \eta^{2} \xi^{\prime \prime } , \\ \end{aligned} $$

27 $$\begin{aligned} & D_{1} (\xi ,\,\xi^{\prime } ,\xi^{\prime \prime } ,\eta ,\,\eta^{\prime } ,\eta^{\prime \prime } ) = s_{2} \xi^{\prime } + s_{3} \eta^{\prime }+ s_{9} \eta \dot{\xi } + s_{10} \xi \dot{\eta } + s_{11} \xi \dot{\xi } \\ & \quad+ s_{12} \eta \dot{\eta }+ s_{15} \xi^{\prime } \eta^{\prime } + s_{22} \xi^{2} \dot{\xi } + s_{23} \eta^{2} \dot{\eta } + s_{24} \eta^{2} \dot{\xi } + s_{25} \xi^{2} \dot{\eta }  , \\ \end{aligned} $$

and

28 $$\begin{aligned} & D_{2} (\xi ,\,\xi^{\prime } ,\xi^{\prime \prime } ,\eta ,\,\eta^{\prime } ,\eta^{\prime \prime } ) = s_{1} \eta^{\prime \prime } + s_{4} \xi + s_{5} \xi \xi^{\prime \prime } + s_{6} \eta \eta^{\prime \prime } + s_{7} \eta \xi^{\prime \prime } + s_{8} \xi \eta^{\prime \prime } + s_{13} \xi^{2} \\ & + s_{14} \eta^{2}  + s_{16} \xi^{3} + s_{17} \eta^{3} + s_{18} \xi^{2} \xi^{\prime \prime } + s_{19} \eta^{2} \eta^{\prime \prime } + s_{20} \eta^{2} \xi^{\prime \prime } + s_{21} \xi^{2} \eta^{\prime \prime } . \\ \end{aligned}$$

As formerly reported^27–30^, $u$ and $v$ ensure that:

29 $$u^{\prime \prime } + \chi_{1} u^{\prime } + \Gamma_{1}^{2} \,\,u = 0,$$

and

30 $$v^{\prime \prime } + \chi_{2} v^{\prime } + \Gamma_{2}^{2} \,\,v = 0,$$

where

31a $$\chi_{1} = \int\limits_{0}^{{2\pi /\varpi_{1} }} {\tilde{u}^{\prime } N_{1} (\tilde{u},\,\tilde{u}^{\prime } ,\tilde{u}^{\prime \prime } ,\tilde{v},\,\tilde{v}^{\prime } ,\tilde{v}^{\prime \prime } )} dt/\int\limits_{0}^{{2\pi /\varpi_{1} }} {\tilde{u}^{\prime 2} \,dt} ,$$

31b $$\,\,\chi_{2} = \int\limits_{0}^{{2\pi /\varpi_{2} }} {\tilde{v}^{\prime } \,D_{1} (\tilde{u},\,\tilde{u}^{\prime } ,\tilde{u}^{\prime \prime } ,\tilde{v},\,\tilde{v}^{\prime } ,\tilde{v}^{\prime \prime } )} dt/\int\limits_{0}^{{2\pi /\varpi_{2} }} {\tilde{v}^{{\prime}{2}} \,dt} ,$$

32a $$\Gamma_{1}^{2} = \int\limits_{0}^{{2\pi /\varpi_{1} }} {\tilde{u}\,N_{2} (\tilde{u},\,\tilde{u}^{\prime } ,\tilde{u}^{\prime \prime } ,\tilde{v},\,\tilde{v}^{\prime } ,\tilde{v}^{\prime \prime } )} dt/\int\limits_{0}^{{2\pi /\varpi_{1} }} {\tilde{u}\,^{2} \,dt} ,$$

and

32b $$\Gamma_{2}^{2} = \int\limits_{0}^{{2\pi /\varpi_{2} }} {\tilde{v}\,D_{2} (\tilde{v},\,\tilde{v}^{\prime } ,\tilde{v}^{\prime \prime } ,\tilde{u},\,\tilde{u}^{\prime } ,\tilde{u}^{\prime \prime } )} dt/\int\limits_{0}^{{2\pi /\varpi_{2} }} {\tilde{v}^{2} \,dt} .$$

Equations (29) and (30) may be transformed to standard simple harmonic motions via the following standard normal forms:

33 $$u(t) = \tilde{u}{\text{Exp}}( - \chi_{1} \,t/2),$$

and

34 $$v(t) = \tilde{v}{\text{Exp}}( - \chi_{2} \,t/2).$$

Thereby,

35 $$\tilde{u}^{\prime \prime } + \varpi_{1}^{2} \tilde{u} = 0,$$

and

36 $$\tilde{v}^{\prime \prime } + \varpi_{2}^{2} \tilde{v} = 0.$$

Consequently, $$\varpi_{1}^{2} = \Gamma_{1}^{2} - \frac{1}{4}\chi_{1}^{2}$$, and $$\varpi_{2}^{2} = \Gamma_{2}^{2} - \frac{1}{4}\chi_{2}^{2}$$.

The stability restrictions can be regarded as:

37 $$\varpi_{1}^{2} > 0,\;{\text{and}}\;\chi_{1} > 0,$$

and

38 $$\varpi_{2}^{2} > 0,\;{\text{and}}\;\chi_{2} > 0.$$

The obtained stability conditions imply that stability is achieved when all eigenvalues of the governing matrix have negative real parts, ensuring that any perturbation at the interface decays with time. Physically, this means that the system naturally suppresses disturbances and prevents oscillations from growing uncontrollably. Instead, excess energy introduced by small perturbations is gradually dissipated through viscous, elastic, and porous resistance mechanisms. Accordingly, the interface is restored to equilibrium, ensuring predictable and well-regulated behavior. From a practical perspective, such stability is essential in both biomedical and industrial applications. In biomedical contexts, it underpins the reliability of microfluidic devices, drug delivery systems, and EHD printing of tissue engineering, where uncontrolled oscillations could compromise precision or safety. Similarly, in industrial processes such as inkjet printing, coating technologies, lubrication systems, and food processing, maintaining stable interfaces is crucial in achieving consistent performance, preventing defects, and ensuring high product quality.

## Validation analysis

### Tabular validation (error analysis and convergence validation)

In what follows, Tables 1 and 2 present a detailed error analysis, comparing the NS of the coupled system given in Eqs. (21)–(22) with the analytical results obtained via NPA in Eqs. (29)–(30). The minimal discrepancies observed confirm the high accuracy and consistency of both methods, highlighting the effectiveness of the proposed approach in modeling complex dynamical systems.

**Table 1:** Comparison of “actual” (numerical) and “approximate” (analytical) solutions at selected times.

Time	Actual “NS”	NPA	Absolute error	Relative absolute error (%)
$0$	$0.2$	$0.2$	$0.0$	$0$
$3$	${ 0}{\text{.199981}}$	$0.2$	${ 0}{\text{.0000185737}}$	${ 0}{\text{.0092877}}$
$6$	${ 0}{\text{.199962}}$	$0.2$	${ 0}{\text{.0000375674}}$	${ 0}{\text{.0187872}}$
$9$	${ 0}{\text{.199943}}$	$0.2$	${ 0}{\text{.0000565593}}$	${ 0}{\text{.0282877}}$
$12$	${ 0}{\text{.199924}}$	$0.2$	${ 0}{\text{.0000755496}}$	${ 0}{\text{.0377891}}$
$15$	${ 0}{\text{.199905}}$	$0.2$	${ 0}{\text{.000094539}}$	${ 0}{\text{.0472918}}$
$18$	${ 0}{\text{.199886}}$	$0.2$	${ 0}{\text{.000113527}}$	${ 0}{\text{.0567956}}$
$21$	${ 0}{\text{.199867}}$	$0.2$	${ 0}{\text{.000132513}}$	${ 0}{\text{.0663004}}$
$24$	${ 0}{\text{.199849}}$	$0.2$	${ 0}{\text{.000151497}}$	${ 0}{\text{.0758061}}$
$27$	${ 0}{\text{.19983}}$	$0.2$	${ 0}{\text{.00017048}}$	${ 0}{\text{.0853128}}$
$30$	${ 0}{\text{.199811}}$	$0.2$	${ 0}{\text{.000189461}}$	${ 0}{\text{.0948205}}$

Errors are small (< 0.1%) and increase gradually with time, consistent with the expected accumulation in reduced-order approximations.

**Table 2:** Comparison of “actual” (numerical) and “approximate” (analytical) solutions at selected times.

Time	Actual “NS”	NPA	Absolute error	Relative absolute error (%)
$0$	$0.2$	$0.2$	$0.0$	$0$
$3$	${ 0}{\text{.199981}}$	$0.2$	${ 0}{\text{.0000183187}}$	${ 0}{\text{.00916022}}$
$6$	${ 0}{\text{.199962}}$	${ 0}{\text{.199999}}$	${ 0}{\text{.0000365468}}$	${ 0}{\text{.0182768}}$
$9$	${ 0}{\text{.199943}}$	${ 0}{\text{.199998}}$	${ 0}{\text{.0000542717}}$	${ 0}{\text{.0271435}}$
$12$	${ 0}{\text{.199924}}$	${ 0}{\text{.199996}}$	${ 0}{\text{.0000715013}}$	${ 0}{\text{.0357642}}$
$15$	${ 0}{\text{.199905}}$	${ 0}{\text{.199994}}$	${ 0}{\text{.0000882469}}$	${ 0}{\text{.0441443}}$
$18$	${ 0}{\text{.199886}}$	${ 0}{\text{.199991}}$	${ 0}{\text{.000104516}}$	${ 0}{\text{.0522878}}$
$21$	${ 0}{\text{.199867}}$	${ 0}{\text{.199988}}$	${ 0}{\text{.000120318}}$	${ 0}{\text{.0601988}}$
$24$	${ 0}{\text{.199848}}$	${ 0}{\text{.199984}}$	${ 0}{\text{.00013566}}$	${ 0}{\text{.0678813}}$
$27$	${ 0}{\text{.199829}}$	${ 0}{\text{.19998}}$	${ 0}{\text{.000150551}}$	${ 0}{\text{.0753396}}$
$30$	${ 0}{\text{.199811}}$	${ 0}{\text{.199976}}$	${ 0}{\text{.000164999}}$	${ 0}{\text{.0825776}}$

Errors are small (< 0.1%) and increase gradually with time, consistent with the expected accumulation in reduced-order approximations.

The tables provide a point sensible comparison of actual (numerical) and approximate (analytical) solutions over the interval $t \in \left[ {0,\,30} \right]$, together with absolute and relative errors. This enables a clear assessment of the accuracy and reliability of the approximation technique while also revealing the temporal evolution of deviations, which may arise from truncation effects, model simplifications, or computational limitations. Such an evaluation is particularly critical in applications such as fluid dynamics, biomedical simulations, and EHD, where even small deviations can significantly affect predictive reliability.

To ensure numerical robustness, convergence was verified by progressively refining temporal resolution until the maximum absolute difference between successive solutions fell below $10^{ - 6}$. Specifically, the maximum absolute error between NS and NPA for $u(t)$ was only $(5 \times 10^{ - 4} )$, with maximum relative error not exceeding 0.0948%. As well, the maximum absolute error between NS and NPA for $v(t)$ was only 0.000343544, with a maximum relative error of 0.0826%. These discrepancies are well below 1%, demonstrating that the analytical approximation closely reproduces the numerical solution. Hence, NPA can be confidently applied as an efficient analytical tool of the present nonlinear model.

The “Actual” values reported in Tables 1, 2 are obtained by direct numerical integration of the full nonlinear coupled system in Eqs. (21) and (22) using MS. The approximate values correspond to reduced linearized model Eqs. (29) and (30), solved under identical conditions. The comparison, therefore, directly validates analytical approximation against a high-accuracy numerical solution of the full nonlinear problem. Representative non-dimensional parameter values used for validation for Tables 1, 2 are:

$$\begin{aligned} & B = 0.2,\,A = 0.2,\,\rho_{3} = 3,\,\rho_{1} = 1,\mu_{1} = .1,\mu_{3} = 2,\,D_{n} = 0.05,\varepsilon_{1} = {0}.002, \\ & \varepsilon_{3} = {0}.005\,,\,P_{e} = 5,\beta_{c} = 2{,}\,a = 0.8, \\ & E_{0} = 0.5,\,\,k = 0.00095,\,\,B_{d} = 10{,}\,{\text{and}}\;Oh = 1.0. \\ \end{aligned}$$

Due to the inherent complexity of the problem, specific simplifications, namely VPF approximation and NPA, are employed. A careful comparison has also been made with relevant studies in the literature that adopt similar methodologies. However, a direct comparison with results derived from solving the full governing equations of fluid motion is not feasible and lies beyond the intended scope of this work.

### Schematic validation

**Fig. 5:**
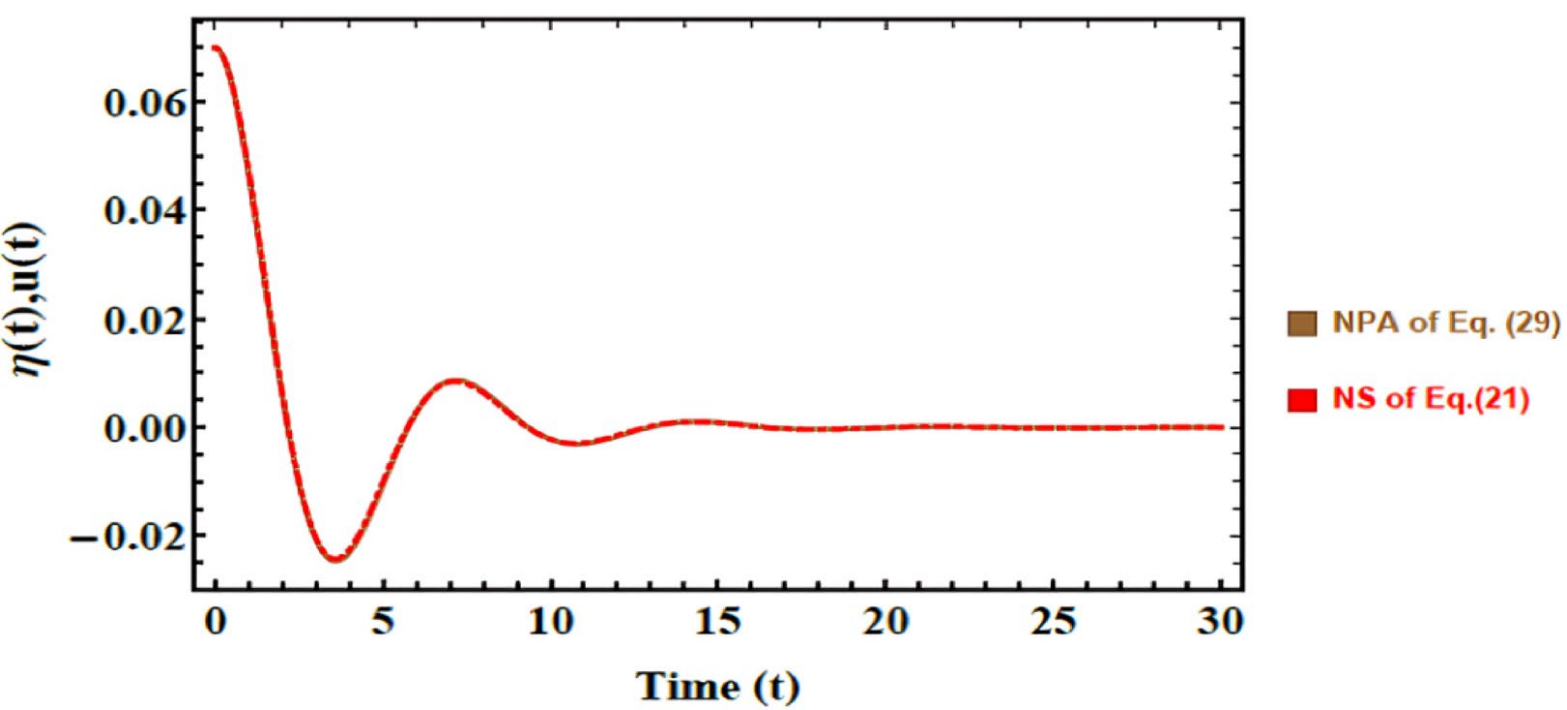
Shows a comparative response between the numerical NS of Eq. (21) and the approximate solution NPA from Eq. (29). The non-dimensional parameters that were held constant are: $$B = 0.09,\,\rho_{3} = 3,\,\rho_{1} = 1,\mu_{1} = 1,\mu_{3} = 2,$$$$A = 0.07,$$ $$D_{n} = 0.5,\varepsilon_{1} = {0}.002,\varepsilon_{3} = {0}.005,$$$$P_{e} = 25,.\beta_{c} = 2{,}\,a = 1.6,$$$$E_{0} = 0.5,\,\,k = 1.15,\,\,B_{d} = 0.005{,}\,\,{\text{and}}\,Oh = 0.022.$$

**Fig. 6:**
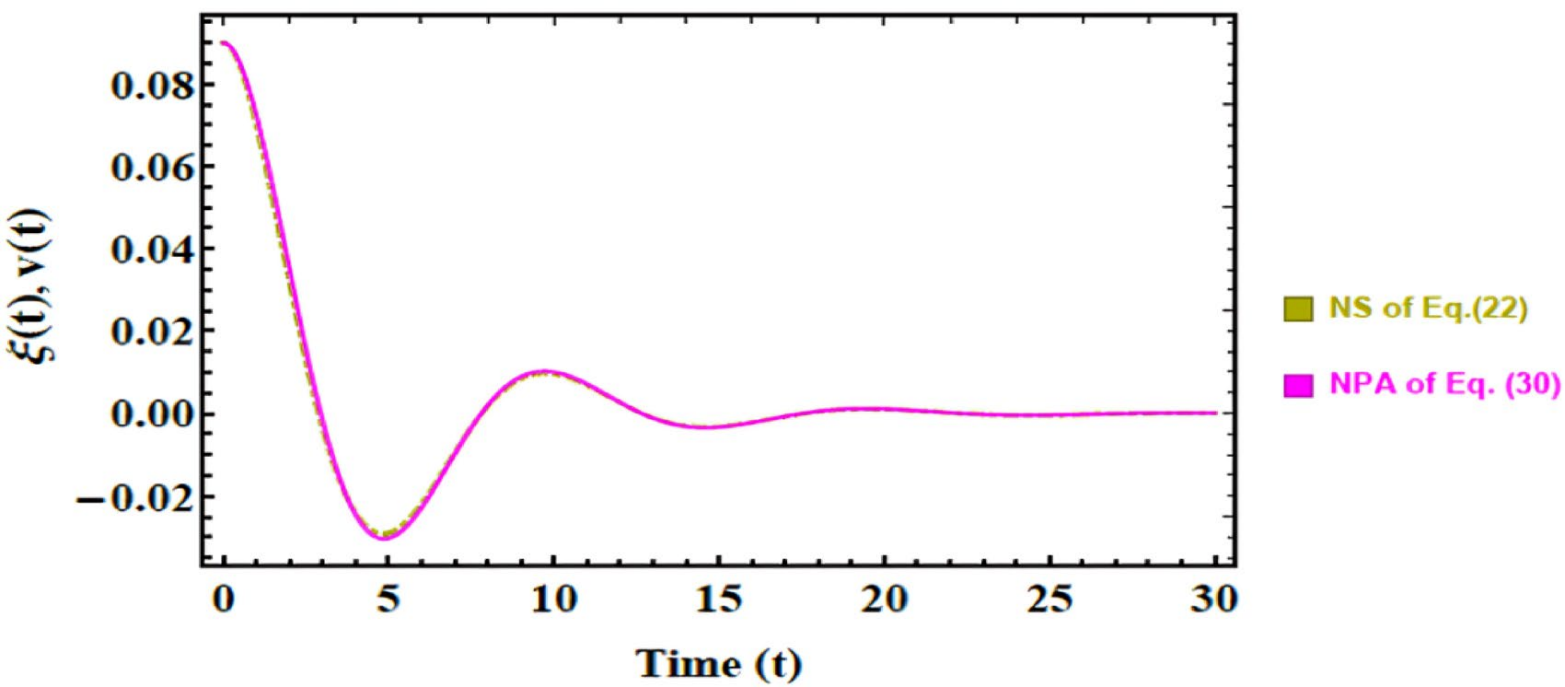
Displays a comparison between the NS of Eq. (22) and the approximate solution NPA from Eq. (30). The non-dimensional parameters that were held constant are as in Fig. 5a, except $\mu_{3} = 3,$ and $P_{e} = 25.$

Figures 5 and 6 provide a direct comparison between NS of nonlinear coupled Eqs. (21) and (22) and the corresponding approximate solutions obtained via NPA, formulated in Eqs. (29) and (30). This comparison highlights the accuracy and effectiveness of NPA in reproducing the nonlinear dynamics of the system. Although primarily introduced as an analytical tool of nonlinear stability analysis, NPA demonstrates broad potential as a reliable and efficient alternative to purely numerical methods in complex physical and engineering contexts.

In Fig. 5, the red curve represents NS of Eq. (21); meanwhile, the brown curve corresponds to NPA solution derived from Eq. (29). The close overlap between the two solutions, with a maximum deviation of only 0.000500803, confirms the high accuracy of NPA in capturing the dynamic response of the system. Similarly, Fig. 6 compares NS of Eq. (22) (yellow curve) with the corresponding NPA result (purple curve). Once again, the two solutions show excellent consistency, with a negligible deviation of just 0.000343544. These minimal discrepancies, well below 1%, underscore the robustness and precision of the developed analytical approach in modeling nonlinear dynamics with reliability.

Finally, the combined evidence from Tables 1, 2 and Figs. 5 and 6 provide a strong validation of the adopted methodology. The excellent agreement between NS and NPA confirms that the developed framework ensures accuracy, convergence, and physical reliability, thereby justifying its use in subsequent stability and parametric investigations.

Beyond numerical accuracy, these results carry clear physical significance. The ability of NPA to realistically reproduce system dynamics ensures that subtle features such as interfacial instabilities and oscillatory damping are correctly predicted. This reliability is critical in practical contexts, where small deviations may lead to large-scale impacts. For instance, in predicting EHD instabilities in biomedical fluids, enhancing drug delivery precision through controlled interfacial motion, and improving adaptive damping strategies in soft robotics and biomedical imaging systems. Accordingly, validation provided by Figs. 5 and 6 not only strengthens the credibility of the present analysis but also confirms the potential of NPA as a practical modeling tool for advanced electrofluidic applications.

A limited situation is examined to validate the current formulation. When Casson and Powell–Eyring parameters are invalidated, the system simplifies to a Newtonian two-layer structure. This classical case was extensively examined in the literature. Our model was directly compared to our prior results^27^. Therefore, the comparison demonstrates a strong concordance with the published analysis.

## Results and discussion

**Fig. 7:**
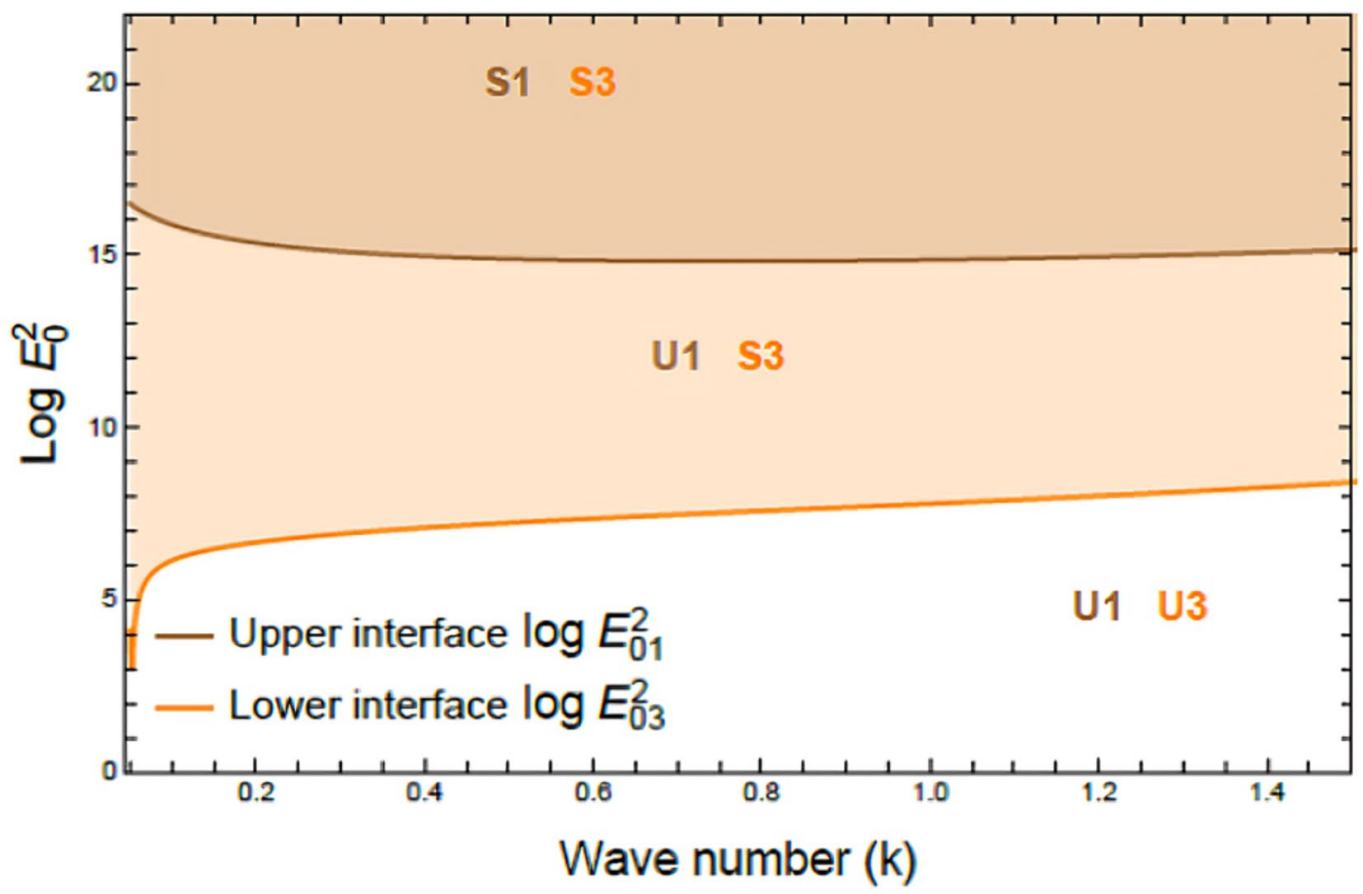
Shows stability boundaries of two liquid interfaces: upper interface $$\log E_{01}^{2}$$ and lower interface $$\log E_{03}^{2}$$ plotted against wave number $$k$$. Stable regions (S1, S3) and unstable regions (U1, U3) are indicated. The non-dimensional parameters held constant: are: $$B = 0.7,\,\,\mu_{3} = 0.0006,\,\,D_{n} = 0.005,$$$$A = 0.7,\,\rho_{3} = 5,\,\rho_{1} = 0.0005,\mu_{1} = 3,$$$$B_{d} = 1.05{,}$$$$\beta_{c} = 0.5{,}\,\,a = 1,$$$$\varepsilon_{1} = {0}.{72},$$$$\varepsilon_{3} = {3}.5\,,\,\,\,P_{e} = 15$$ and $$Oh = 2.05$$.

**Fig. 8:**
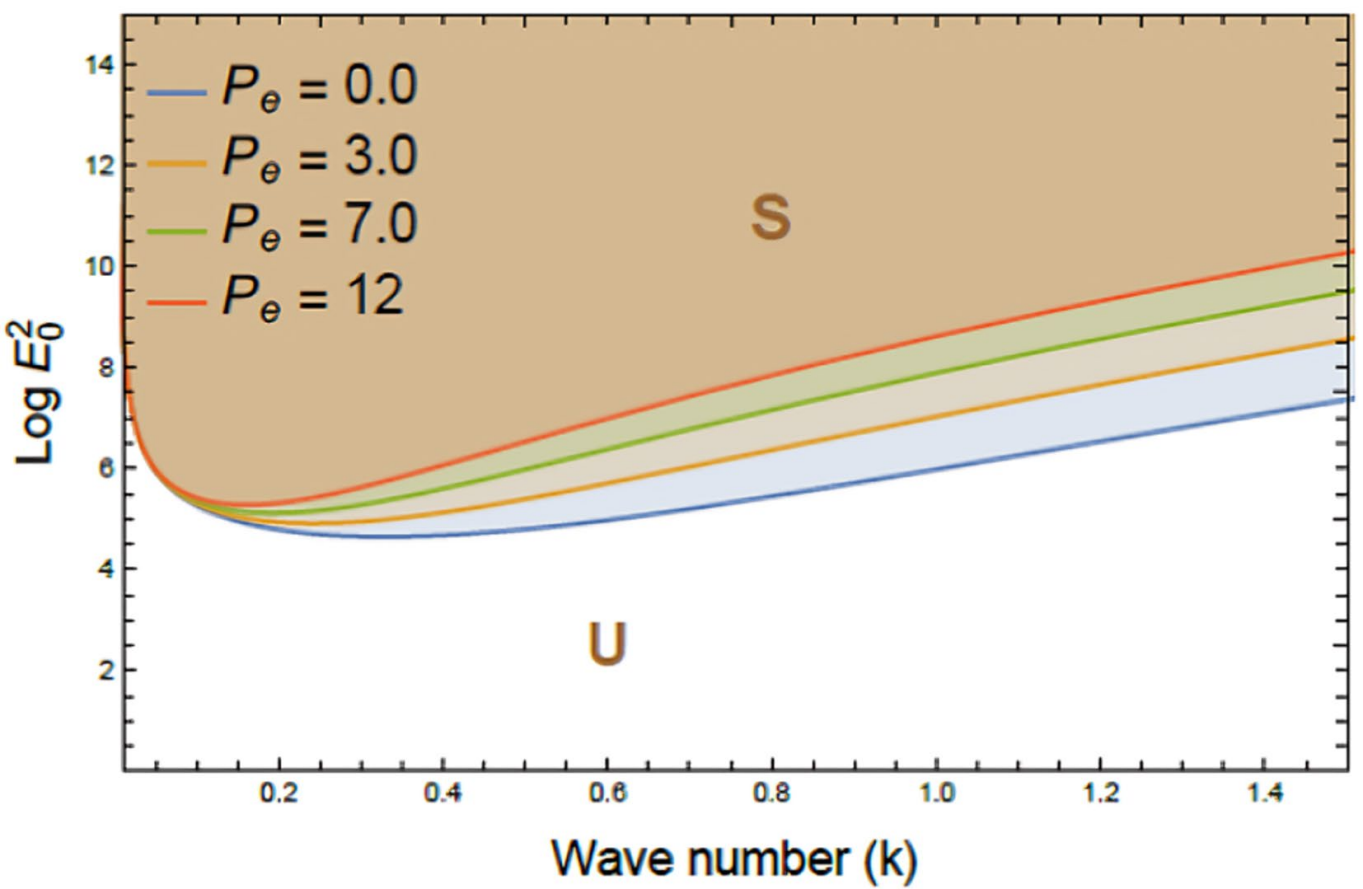
Displays the effect of PEL number $P_{e}$ on stability boundaries $(\log E_{0}^{2} \,{\text{vs}}{.}\,\,k)$, $P_{e}$ varies from 0.0 to 12, while other parameters remain fixed. Stable (S) and unstable (U) regions are indicated. The non-dimensional parameters held constant are as in Fig. 7, except $D_{n} = 1.00$.

**Fig. 9:**
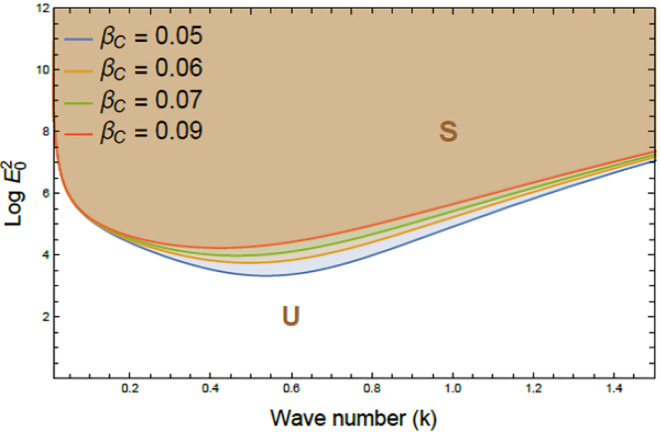
Demonstrates the impact of the CL factor $\beta_{c}$ on stability boundaries $(\log E_{0}^{2} \,{\text{vs}}{.}\,\,k)$ $\beta_{c}$ rises from 0.05 to 0.09, meanwhile other parameters remain fixed. Stable (S) and unstable (U) regions are indicated. The non-dimensional parameters held constant are as in Fig. 7, except $D_{n} = 1.005,$ and $P_{e} = 0.5$

**Fig. 10:**
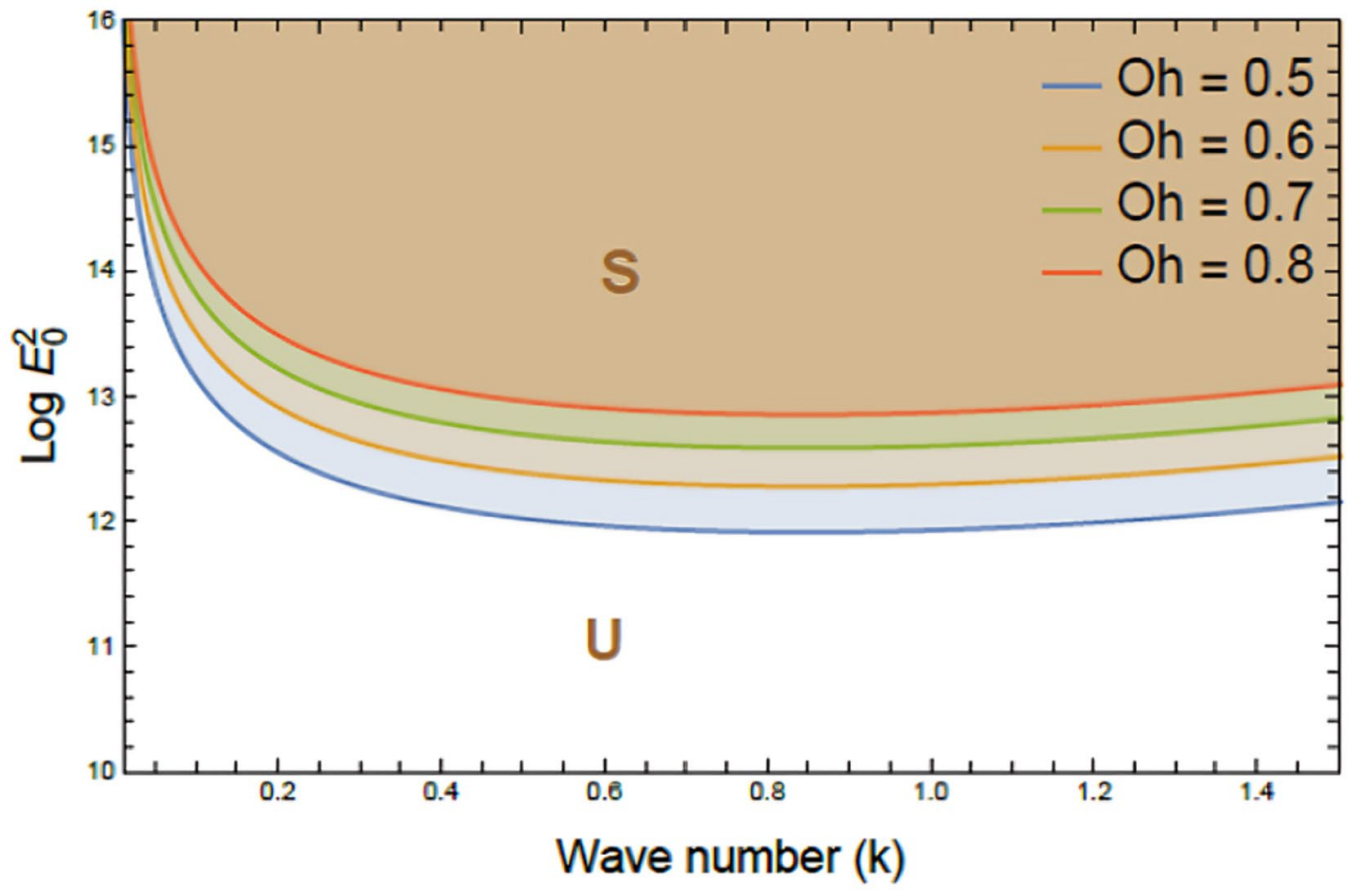
Depicts the influence of the Ohnesorge number $Oh$ on stability boundaries $(\log E_{0}^{2} \,{\text{vs}}{.}\,\,k)$, $Oh$ changes from 0.5 to 0.8, meanwhile other parameters remain fixed. Stable (S) and unstable (U) regions are indicated. The non-dimensional parameters held constant are as in Fig. 7, except $P_{e} = 10$.

**Fig. 11:**
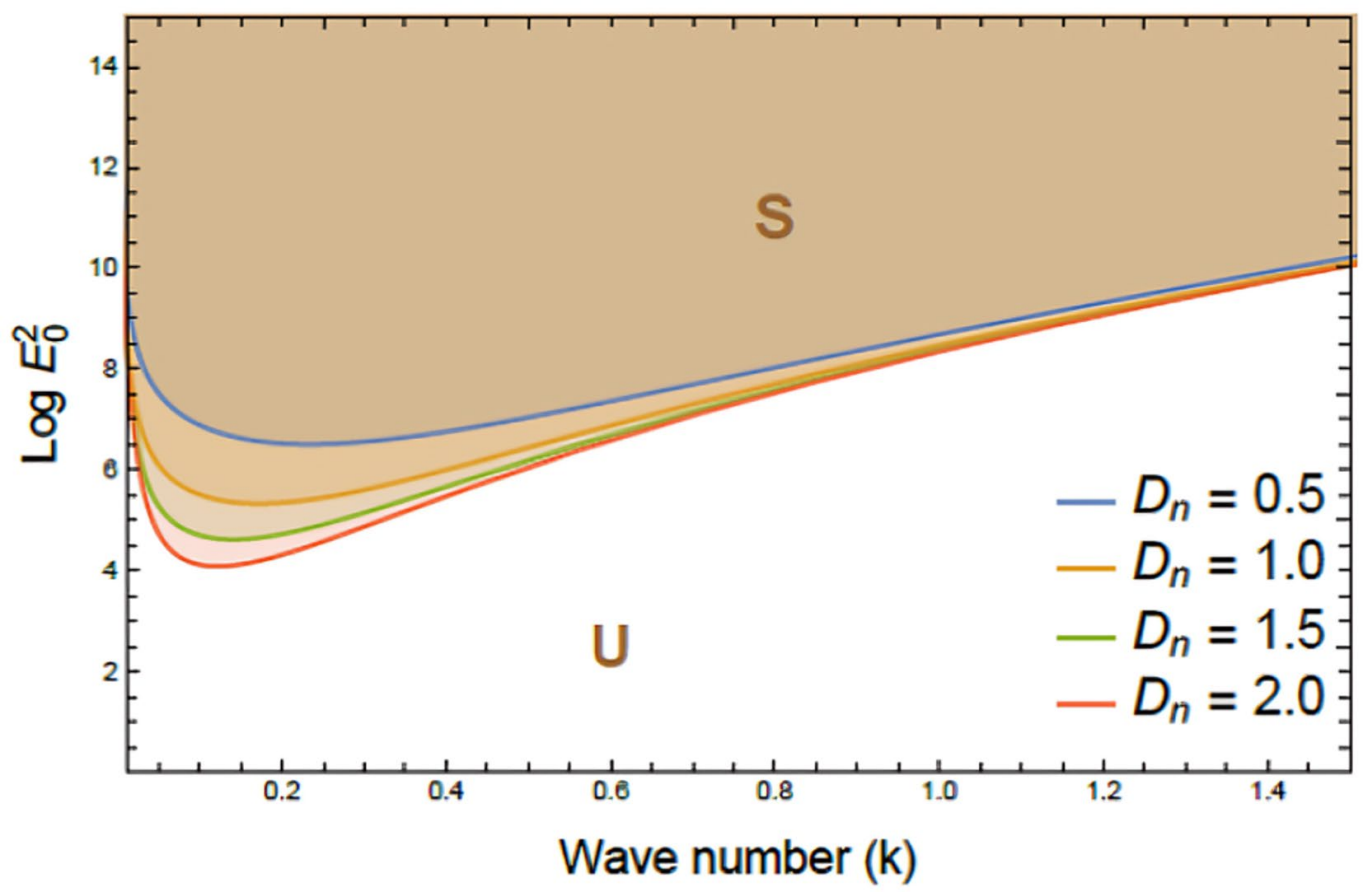
Exhibits variation of stability boundaries $(\log E_{0}^{2} \,{\text{vs}}{.}\,\,k)$ with Darcy number $D_{n}$_._ Curves plotted for diverse values of $D_{n}$, while other parameters remain fixed. Stable (S) and unstable (U) regions are indicated. The non-dimensional parameters held constant are as Fig. 7, except $P_{e} = 10$.

**Fig. 12:**
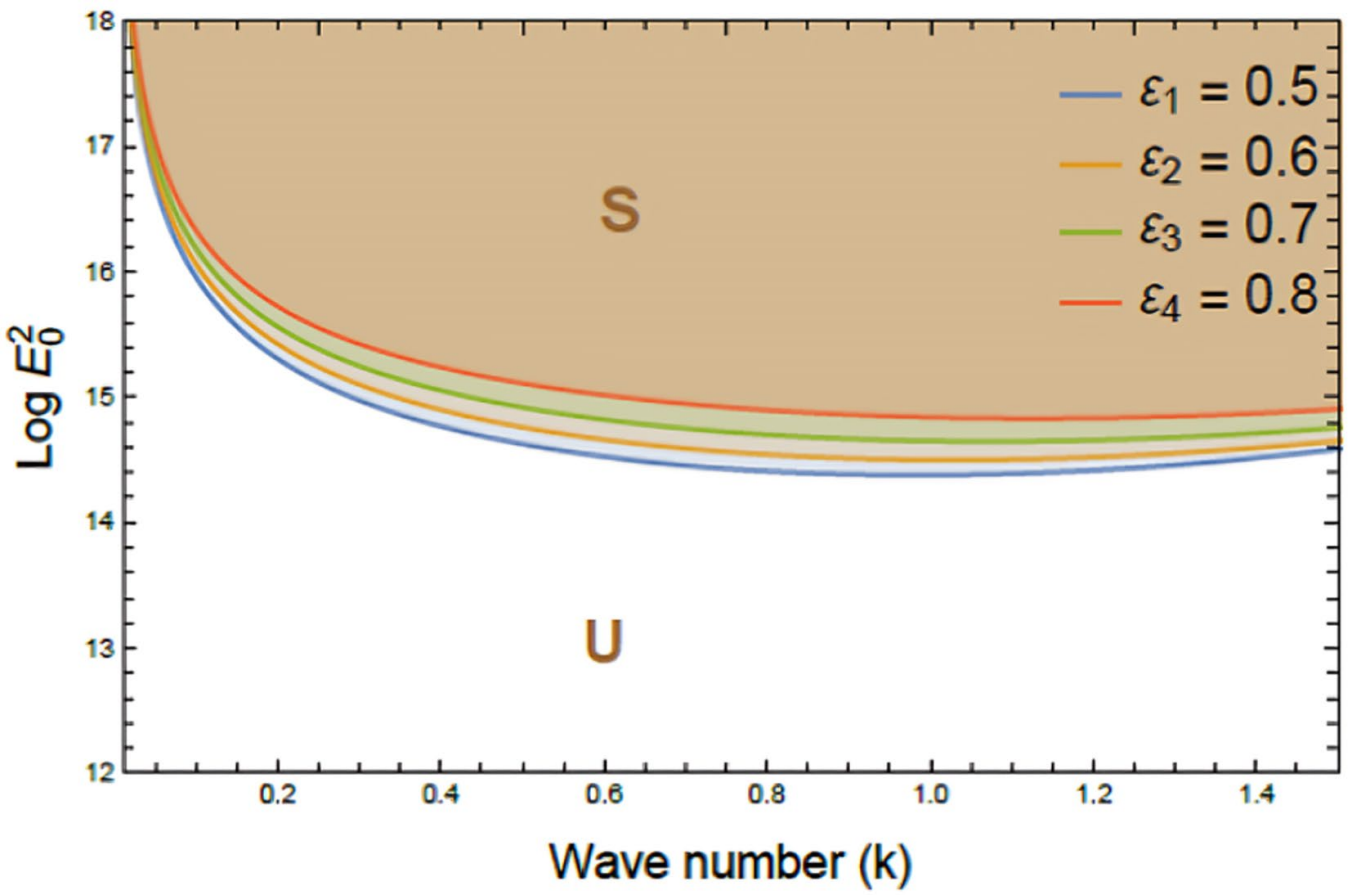
Reflects the effect of dielectric constant $\varepsilon_{1}$ on stability boundaries $(\log E_{0}^{2} \,{\text{vs}}{.}\,\,k)$. Curves show $\varepsilon_{1}$ increasing progressively from 0.5 to 0.8; meanwhile, other parameters remain fixed. Stable (S) and unstable (U) regions are indicated. The non-dimensional parameters held constant are as in Fig. 7, except $\varepsilon_{3} = {1}.5\,,\,$ and $P_{e} = 10$.

Figures 7, 8, 9, 10, 11, 12 depict the stability charts based on the constraints defined in Eqs. (37) and (38). These constraints include essential parameters such as PEL numeral $P_{e}$, CL factor $\beta_{c}$, Darcy numeral $D_{n}$, dielectric constant $\varepsilon_{1}$, and Ohnesorge numeral $Oh$. The frequencies$\varpi_{1}^{2}$ &$\varpi_{2}^{2}$, and initial amplitudes $A$ & $B$, are also included to reflect the system’s stability behavior. The darker regions above each curve point out stable regions in these plots. Conversely, lighter zones below curves reflect regions of instability. Each subfigure investigates how variations in a specific parameter influence the system’s response. The horizontal axis typically shows wave numeral $k$; meanwhile, vertical axis reflects EF number, often presented in logarithmic form $\log E_{0}^{2}$. These graphs reveal how changes in physical forces impact the balance of stability and instability in the interface. The physical interpretation lies in the interaction between competing forces, capillary tension, EF influences, and viscous or inertial contributions. These forces together shape the dynamic movement and deformation of the liquid interface. Collectively, dimensionless parameters utilized in this analysis encapsulate the essential physical performances of the liquid system, enabling a generalized interpretation of its performance across diverse spatial scales and engineering applications.

In a horizontal plane configuration comprising three immiscible liquid layers, Fig. 7 examines the stability profile of two interfacial boundaries by plotting $\log E_{0}^{2}$ versus $k$ to describe stable and unstable areas. The three non-Newtonian fluids are stratified, forming dual interfaces: the upper interface, between the first and second liquids, is described by the function $\log E_{01}^{2}$ , meanwhile the lower interface, between the second and third fluids, is expressed by $\log E_{03}^{2}$. These curves reflect the dynamic response of each interface to small disturbances. Stability regions of upper and lower interfaces are identified as S1 and S3, respectively, whereas the corresponding unstable zones are symbolized as U1 and U3. The regions above the curve $\log E_{01}^{2}$ exhibit more uniform and smoother stability profiles compared to those associated with $\log E_{03}^{2}$, indicating that the outer (upper) interface exerts a more significant influence on the system’s global stability. This performance recommends that $\log E_{01}^{2}$ acts as the dominant curve governing the system’s overall dynamical conduct. The analysis further incorporates influences of nonlinear interactions between the interfaces, accounting for the influence of critical dimensionless numerals such as Darcy, PEL, and Ohnesorge numbers. These quantities collectively modulate interfacial response, shaping boundaries between stability and instability across the system^30^.

Figure 8 reflects the influence of PEL numeral $P_{e}$ on stability boundaries of the system, outlined by the variation of $\log E_{0}^{2}$ against $k$. As the PEL numeral enlarges from 0.0 to 12, a notable expansion of the unstable area U is noticed; meanwhile, the stable zone S diminishes correspondingly. This shift emphasizes a pronounced destabilizing conduct imparted by non-Newtonian rheological performance captured by PEL. From a physical view, the PEL number reflects the extent of shear-thinning conduct in non-Newtonian fluids, where viscosity diminishes with escalating shear rate. As PEL rises, the fluid becomes more sensitive to velocity gradients, reducing viscous damping and weakening the system’s resistance to disturbances, thus promoting instability. This performance stems from a shift in balance between inertia and internal resistance; at high PEL amounts, inertia prevails, allowing interfacial waves to grow unchecked. Practically, the destabilizing impact of high PEL is critical in electrofluid applications. It can disrupt drug delivery, cause turbulence in microcirculation, and impair stability in EHD devices. Managing PEL is essential in ensuring uniform inflow and system reliability^33^.

Figure 9 highlights the influence of the CL factor on nonlinear stability characteristics of the system, as depicted by the relationship between $\log E_{0}^{2}$ and $k$. As the CL factor escalates from 0.05 to 0.09, the plotted curves exhibit a noticeable upward shift in the stability zone. This indicates a contraction in stable region S and a corresponding expansion of unstable one U, underscoring the system’s increased susceptibility to disturbances. Physically, higher CL values reduce effective viscosity beyond yield stress, steepening velocity gradients and lowering damping, which enhances instability under EFs. Practically, this matters in EHD systems like inkjet printing, microfluidics, and biomedical flows, where elevated CL can cause irregular flow or wave growth. Managing CL is the key to maintaining precision and stability in such applications^37^.

Figure 10 demonstrates the destabilizing impact of the Ohnesorge numeral $Oh$ on nonlinear stability that characterizes viscoelastic electron fluids, as depicted by the relationship between $\log E_{0}^{2}$ and $k$. The plotted curves correspond to escalating $Oh$ amounts (0.5, 0.6, 0.7, and 0.8), exhibiting a consistent upward shift in the critical stability zone. This progression reveals that as $Oh$ improvements, stable region S contracts significantly, meanwhile unstable domain U broadens across the entire wave numeral spectrum. In a physical context, increased viscosity disturbs the equilibrium between shear-thinning and inertia, resulting in reduced damping. This renders the system more susceptible to instabilities, particularly under intense EFs in micro-scale environments. This is critical of EHD applications like electro-spraying, droplet formation, and lab-on-chip devices. In biomedical systems, high viscosity may destabilize drug droplets or hinder influx in synthetic biofluids, affecting treatment or sensor function. Managing this influence is a key to stable performance in high-viscosity, electrically driven systems^27^.

Figure 11 underlines the influence of Darcy numeral $D_{n}$ on the nonlinear stability of a two-plane interface system within viscoelastic electrofluids, by depicting the variation of $\log E_{0}^{2}$ against $k$. The curves correspond to different $D_{n}$ measures that escalate, and curves shift downward, signifying a decrease in the critical threshold of stability. Consequently, S becomes broader, meanwhile U is progressively diminished, indicating a pronounced stabilizing performance as permeability enriches. Physiologically, the Darcy number outlines the permeability of a porous medium. A higher amount indicates greater permeability, allowing smoother fluid influx and enhanced energy dissipation, which helps dampen disturbances and delay interfacial instability. This has important applications in EHD systems involving porous media, such as filtration, tissue fluid transport, and microfluidics. In biomedical engineering, optimizing Darcy numbers aids in modeling fluid inflow through tissue scaffolds or drug diffusion in membranes. In geophysics, it informs subsurface flow predictions, while in industry, tuning it improves stability in systems like porous electrodes^27,28^.

Figure 12 clarifies the performance of the dielectric constant $\varepsilon_{1}$ on nonlinear stability characteristics of two-plane interfaces in viscoelastic electrofluids. The plot shows variation of $\log E_{0}^{2}$ vs $k$. As the dielectric constant $\varepsilon_{1}$ escalates, stability curves shift downward, thereby shrinking S and expanding U. This pattern clearly indicates a destabilizing influence of higher dielectric contrast within the system. From a physical standpoint, the dielectric constant determines how strongly a liquid responds to an EF through polarization. Higher values increase electric stresses at the interface, promoting deformation and dwindling stability thresholds. This enhances the risk of instability by lowering the energy barrier of perturbation growth. In EHD systems, such as microfluidics, drug delivery, inkjet printing, and liquid lenses, elevated dielectric constants can improve actuation but also heighten susceptibility to interfacial breakdown. Consequently, controlling dielectric properties is crucial in maintaining system stability and performance^27,28^.

In what follows, Table 3 summarizes complex interactions among key physical parameters and their impact on the stability of viscoelastic electrofluid interfaces under EFs. Each parameter represents a distinct mechanistic pathway governing interfacial dynamics.

**Table 3:** Key factors influencing fluid stability.

Parameter	Performance	Physical Mechanism	Practical applications
$P_{e}$	Elevated standards promote interfacial destabilization	Escalates non-Newtonian shear-thinning conduct, leading to intensified velocity gradients and diminished viscous damping	Triggers inflow irregularities in blood vessels, disrupts controlled drug dispersion, and affects nutrient transport across biological membranes
$\beta_{c}$	Increasing measures tend to destabilize liquid interfaces	Elevates yield stress threshold, causing delayed inflow initiation and nonlinear resistance to deformation	Critical in modeling blood rheology, influencing polymer solution inflows, and regulating resistance in tissue-engineered microfluidics
$Oh$	Higher amounts reduce interface stability	A lower viscous-to-inertial ratio limits damping, enhancing the amplitude of surface-tension-induced oscillations	This leads to droplet breakup in drug delivery systems, instability in respiratory mucus layers, and inaccuracies in biomedical inkjet printing
$D_{n}$	Increased amounts enhance interface stability	Higher permeability diminishes internal liquid resistance, dampening convective inflows and suppressing perturbations	Pivotal for optimizing perfusion in porous tissues, controlling transdermal drug delivery, and maintaining uniform inflow in biological scaffolds
$\varepsilon_{1}$	Amplifies destabilization with escalated measures	Enlarges EHD stresses by escalating charge accumulation at interfaces, leading to stronger normal stress imbalances	Influences electrofluidic precision, affects interface stability in lab-on-chip systems, and aids in the electrophoretic control of biological particles

**Fig. 13:**
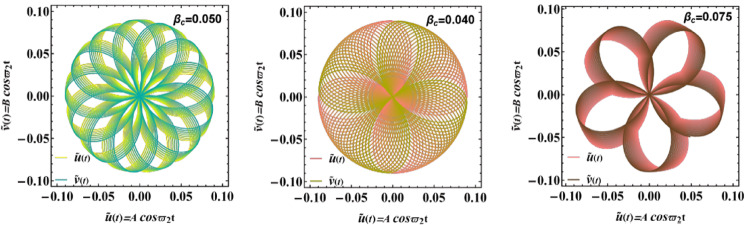
Displays a Polar representation of trajectories showing the variation of $$\tilde{u}\left( t \right) = A\cos \varpi_{2} t$$ vs. $$\hat{v}(t) = B\cos \varpi_{2} t$$ influenced by $$\beta_{c}$$. The non-dimensional parameters held constant: are: $$A = 0.09,\,\rho_{3} = 3.0005,\,\rho_{1} = 1.0005,\mu_{1} = 1.0005,$$ $$B = 0.09,\,\,\mu_{3} = 2.0006,\,\,D_{n} = 0.5,$$$$B_{d} = 1.05{,}$$$$\varepsilon_{1} = {0}.{00}02,$$$$a = 1.6,$$$$\varepsilon_{3} = 0.0005\,,\,P_{e} = 10$$ and $$Oh = 0.15$$.

Figure 13 presents polar trajectories that display the relationship of $\tilde{u}\left( t \right) = A\cos \varpi_{2} t$ vs. $\hat{v}(t) = B\cos \varpi_{2} t$ of diverse amounts of the Casson factor $\beta_{c}$. The influence of the Casson parameter $\beta_{c}$ on the system’s dynamics is clearly reflected in polar phase portraits. For smaller amounts of $\beta_{c}$, trajectories are tightly wound and nearly circular, indicating that oscillatory behavior remains close to Newtonian dynamics with regular and symmetric displacement patterns. As $\beta_{c}$ escalates, however, the role of yield stress becomes dominant, introducing stronger nonlinearities into motion. This shift is visible through the development of lobed or flower-like trajectories, which signify partial energy trapping and a departure from purely harmonic responses. From a physical standpoint, this performance arises from additional resistance in Casson-type fluids once the yield threshold is exceeded, which alters the balance between inertia and damping. Such insights are highly relevant in biomedical applications, particularly in modeling blood flow, as well as in engineering contexts such as electrofluidic devices and micro-actuators, where precise control of non-Newtonian interfaces is required^27,30^.

**Fig. 14:**
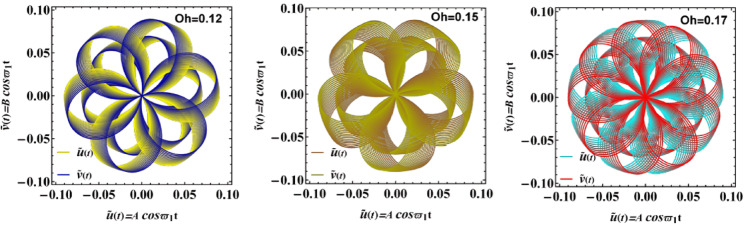
Expounds a Polar diagram of trajectories showing the relationship between $$\hat{u}(t) = A\cos \varpi_{1} t$$ and $$\hat{v}(t) = B\cos \varpi_{1} t$$ impacted by $$Oh$$. The non-dimensional parameters held constant: are:$$A = 0.07,\,\rho_{3} = 3.0005,\,\rho_{1} = 1.0005,\mu_{1} = 1.0005,$$ $$P_{e} = 10$$$$B = 0.09,\,\,\mu_{3} = 2.0006,\,\,D_{n} = 0.57,$$$$B_{d} = 0.005{,}$$$$\beta_{c} = 2{\text{,a}}$$$$a = 0.62,$$$$\varepsilon_{3} = 0.0005\,,$$ and $$\varepsilon_{1} = {0}{\text{.0002}}$$.

Figure 14 highlights the polar diagrams between $\hat{u}(t) = A\cos \varpi_{1} t$ $,$ and $\hat{v}(t) = B\cos \varpi_{1} t$ of varying Ohnesorge numeral $Oh = 0.12,\,0.15,\,0.17$, which quantify the influence of viscous damping relative to inertial and capillary forces in fluid systems. The effect of the Ohnesorge number $Oh$ on system response is illustrated in polar phase portraits. For relatively small $Oh$ amounts (e.g., $Oh = 0.12$), trajectories display sharp, multi-lobed structures, which reflect predominance of inertial and surface tension influences over viscous damping. As $Oh$ enlarges to moderate values (e.g., $Oh = 0.15$), phase trajectories become smoother and more symmetric, signalling growing influence of viscosity in moderating oscillations. At higher $Oh$ (e.g., $Oh = 0.17$), trajectories exhibit diffused, intertwined patterns, where viscous effects dominate and energy dissipation reduces the regularity of oscillatory motion. From a physical standpoint, this transition highlights the balance between inertia and viscosity: lower $Oh$ values favour pronounced oscillations with higher energy retention, while higher $Oh$ amounts promote damping and suppression of oscillatory instabilities. These insights are particularly relevant to applications involving droplet dynamics, inkjet printing, spray cooling, and biomedical microfluidics, where precise control over the interplay between viscous, inertial, and surface tension forces is essential for stability and performance. From a physical standpoint, this trend emphasizes the central role of liquid viscosity in shaping the nonlinear behavior of interfacial motion, especially in multilayer systems. Such understanding is vital in EHD and microfluidic applications. Examples include lab-on-chip devices, droplet generation, and biomedical actuators. In these systems, interface damping must be carefully managed. The Ohnesorge number provides a useful tool in improving fluid control in non-Newtonian or multiphase liquids under electrostatic influence^27–30^.

From an application standpoint, these results inform design choices: for example, to avoid interfacial mixing or unwanted oscillations in microfluidics, one would operate in parameter windows that produce small, symmetric loops (low higher-harmonic content and strong damping); conversely, if one needs enhanced interfacial mixing, operating near parameters that produce lobed, nonlinear trajectories may be advantageous.

## Concluding thoughts

The analysis of nonlinear stability in double horizontal interfaces segmented by three-stratified non-Newtonian liquids is crucial for advanced management, microfluidics, and coating technologies, as it explores unique thermal and rheological properties. The incorporation of non-Newtonian liquids demonstrates distinct rheological characteristics, requiring an in-depth analysis of their impact on interfacial instabilities in multilayer systems. The central component included a CL; meanwhile, the upper and lower regions were occupied by PELs. The influence of an unchanged tangential EF and surface tension was examined in porous media. VPF was employed to enable mathematical applications. The problem chiefly concerned the integration of fundamental hydrodynamic governing equations with Maxwell’s equations in a quasi-static approach. The linearized control equations were developed to obtain a nonlinear expression under designated nonlinear BCs. The impacts of viscoelasticity were accordingly neglected in the solution of equations of motion. Consequently, the planar axis and interface perturbation were interacting laterally. HFF converted traditional nonlinear ODEs into linear ones, which were analyzed by NPA. The non-dimensional physical properties can be employed to analyze fundamental characteristics of a liquid system. Moreover, they lowered the number of qualities necessary to understand the structure. An abridged summary of the NPA was provided. The numerical simulations demonstrated that the entire system was stabilized by different configurations of tangential EF in relation to horizontal wavenumber. Polar diagrams were designed via the PolarPlot command to elucidate the impacts of diverse causes and ensure the consistency of replies. The core outcomes derived from this investigation are outlined as follows:

1. The developed model, consisting of a pair of coupled nonlinear ODEs, effectively captures the dynamics of a dual-interface system with two degrees of freedom.
2. Numerical simulations demonstrated good agreement between NPA and NS outcomes, supported by structured comparisons through tables and schematic visualizations.
3. Certain parameters exhibited a destabilizing influence; meanwhile, an escalation in Darcy number significantly improved stability.
4. PolarPlot visualizations revealed the influence of various physical parameters on system performance, with unstable solutions excluded from plots to ensure physical relevance.

### Limitations

1. The first consideration concerns the application of VPF, which treats viscous or viscoelastic liquids as idealized flows. By imposing ideal inflow constraints, the method enables the construction of a relatively simple mathematical expression to represent the time-dependent displacement of the interface. Without this assumption, governing equations would become considerably more complex, demanding advanced and computationally intensive solution techniques. Consequently, adopting ideal inflow constraints is essential, as it allows the elimination of pressure terms from the momentum equation via Bernoulli’s principle, thereby simplifying the analysis and improving tractability. Additionally, the theory of VPF, which enhances classical potential flow by incorporating viscous normal stresses while disregarding vorticity, becomes progressively unsuitable at elevated Reynolds numbers due to the predominance of thin boundary layers, shear layers, and turbulence, where vorticity and nonlinear viscous effects are essential.
2. The second consideration involves the choice of initial amplitude in NPA. To maintain a reasonable level of accuracy when comparing the nonlinear and linear ODEs, the initial perturbation amplitude must remain small, specifically less than unity. If the amplitude is too large, the validity of the linear approximation deteriorates, causing divergence between nonlinear and linear models. Therefore, constraining the initial amplitude ensures that the linear model remains a reliable representation of the system’s behavior under perturbation.

### Future work

Investigating the nonlinear stability of double interfaces subjected to periodic EFs is crucial, as these systems occur in various physical and technical domains, such as EHD fluxes, microfluidics, thin film technologies, and plasma confinement. Double interfaces create intricate mode interactions, wherein instabilities may pair and develop nonlinearly, resulting in diverse dynamical phenomena such as pattern generation, resonance, or instability suppression, contingent upon frequency and amplitude of applied EF. Comprehending these nonlinear dynamics is crucial in forecasting long-term system behavior, regulating interface morphology, and engineering devices that leverage or alleviate instability consequences. Furthermore, periodic forcing induces parametric resonances that may either stabilize or destabilize the interfaces, rendering this study essential for the advancement of theoretical models and practical applications dependent on precise interface regulation.

## Supplementary Information

Below is the link to the electronic supplementary material.

Supplementary Material 1

## Data Availability

All data generated or analyzed during this study are included in this published article.

## Abbreviations

CL: Casson liquid
PEL: Powell–Eyring liquid
EF: Electric field
VPF: Viscous potential flow
ODE: Ordinary differential equation
HFF: He’s frequency formula
NPA: Non-perturbative approach
EHD: Electrohydrodynamics
RTI: Rayleigh–Taylor instability
KHI: Kelvin–Helmholtz instability
HPM: Homotopy perturbation method
BCs: Boundary conditions
ICs: Initial conditions

## List of symbols

$$A\,,\,B$$: Initial amplitudes
$$B_{d}$$: Bond numeral
$c.c.$: Complex conjugate of former terms
$$D_{n}$$: Darcy numeral
$P_{e}$: Powell–Eyring factor
$\underline{e}_{x}$: Unit vector along the *x*-axis
$\underline{e}_{y}$: Unit vector along the *y*-axis
$$\underline {g}$$: Gravitational acceleration (L T^−2^)
$$E_{0}^{2}$$: Electric intensity
$$k$$: Wave numeral (L^−1^)
$\underline{n}_{{a,(-a)}}$: Two interface units outward normal
$$p_{j}$$: Hydrostatic pressure $$({\text{Newton/L}}^{2} )$$
$$p_{y}$$: Yield stress
$$S$$: Stable area
$S_{{a,\;(-a)}}$: Surface profile of interfaces
$$t$$: Time $$({\text{T}})$$
$$U$$: Unstable area
$$\underline{V}_{j}$$: Liquid velocity (L T^−1^)
$$(x,y)$$: Cartesian coordinates
$$Oh$$: Ohnesorge numeral

## Greek symbols

$$\alpha$$: Darcy’s coefficients (M L^−3^ T^−1^)
$$\beta_{c}$$: Casson factor
$\varsigma ,\,\gamma$: Powell–Eyring constants
$$\delta_{ij}$$: Kronecker delta
$$\varepsilon_{j}$$: Dielectric constants
$$\mu_{j}$$: Dynamic viscosities (M L^−1^ T^−1^)
$$p_{j}$$: Liquid densities (M L^−3^)
$\sigma_{ij}^{total}$: Total stress tensor
$\sigma_{ij}^{hydro}$: Viscous stress tensor
$\sigma_{ij}^{electro}$: Electric stress tensor
$\eta ,\,\xi$: Surface displacement of interfaces (L)
$\pi_{c}$: Critical deformation rate amount
$\varphi_{j}$: Potential function of velocities (L^2^ T^−1^)
$\psi_{j}$: Electric potential (Ampere)
$$\varpi_{1,2}$$: Total frequencies

## References

[1] Gonzalez, A. & Castellanos, A. Nonlinear waves in a viscous horizontal film in the presence of an electric field. *J. Electrostat.* **40–41**, 55–60 (1997).

[2] Rudraiah, N., Krishnamurty, B. S. & Mathad, R. D. The effect of an oblique magnetic field on the surface instability of a finite conducting fluid layer. *Acta Mech.* **119**, 165–180 (1996).

[3] El-Sayed, M. F. Electrohydrodynamic interfacial stability conditions in the presence of heat and mass transfer and oblique electric fields. *Z. Naturforsch.* **54a**, 470–476 (1999).

[4] Melcher, J. R. *Field Coupled Surface Waves* (MIT Press, 1963).

[5] Awasthi, M. K. Electrohydrodynamic capillary instability with heat and mass transfer. *Ain Shams Eng. J.* **5**(1), 263–270 (2014).

[6] Li, F., Ozen, O., Aubry, N., Papageorgiou, D. T. & Petropoulos, P. G. Linear stability of a two-fluid interface for electrohydrodynamic mixing in a channel. *J. Fluid Mech.* **583**, 347–377 (2007).

[7] Moatimid, G. M. & Obied Allah, M. H. Electrohydrodynamic linear stability of finitely conducting flows through porous fluids with mass and heat transfer. *Appl. Math. Model.* **34**(10), 3118–3129 (2010).

[8] Smorodin, B. L. & Kartavykh, N. N. Periodic and chaotic oscillations in a low conducting liquid in an alternating electric field. *Microgravity Sci. Technol.* **32**(3), 423–434 (2020).

[9] Li, P. et al. Heat transfer of hybrid nanomaterials base Maxwell micropolar fluid flow over an exponentially stretching surface. *Nanomaterials* **12**(7), 1207 (2022). 35407325 10.3390/nano12071207 PMC9000894

[10] El-Sayed, M. F. Hydromagnetic instability conditions for viscoelastic non-Newtonian fluids. *Z. Naturforsch.* **55a**, 460–466 (2020).

[11] Su, Y. Y. & Khomami, B. Purely elastic interfacial instabilities in superposed flow of polymeric fluids. *Rheol. Acta* **31**, 413–420 (1992).

[12] Alves, M. A., Oliveira, P. J. & Pinho, F. T. Benchmark solutions for the flow of Oldroyd-B and PTT fluids in planar contractions. *J. Nonnewton. Fluid Mech.* **110**(1), 45–75 (2003).

[13] Sirwah, M. A. Linear instability of the electrified free interface between two cylindrical shells of viscoelastic fluids through porous media. *Acta. Mech. Sin.* **28**(6), 1572–1589 (2012).

[14] Lamb, H. *Hydrodynamics* 6th edn. (Cambridge University Press, 1932).

[15] Funada, T. & Joseph, D. D. Viscous potential flow analysis of Kelvin–Helmholtz instability in a channel. *J. Fluid Mech.* **445**, 263–283 (2001).

[16] Funada, T. & Joseph, D. D. Viscous potential flow analysis of capillary instability. *Int. J. Multiph. Flow* **28**(9), 1459–1478 (2002).

[17] Feng, X., Chen, X. B. & Dias, F. A potential flow model with viscous dissipation based on a modified boundary element method. *Eng. Anal. Bound. Elem.* **97**, 1–15 (2018).

[18] Sánchez-Caja, A., Martio, J. & Siikonen, T. A coupled potential–viscous flow approach for the prediction of propeller effective wakes in oblique flow. *J. Mar. Sci. Technol.* **24**(3), 799–811 (2019).

[19] Zhang, L., Zhang, J.-N. & Shang, Y.-C. A Potential flow theory and boundary layer theory based hybrid method for water jet propulsion. *J. Mar. Sci. Technol.* **7**(4), 113 (2019).

[20] El-Sayed, M. F., Eldabe, N. T., Haroun, M. H. & Mostafa, D. M. Nonlinear Kelvin-Helmholtz instability of Rivlin-Ericksen viscoelastic electrified fluid-particle mixtures saturating porous media. *Eur. Phys. J. Plus* **127**, 29 (2012).

[21] He, J.-H. Homotopy perturbation technique. *Comput. Methods Appl. Mech. Eng.* **178**, 257–262 (1999).

[22] Salas, A. H. & El-Tantawy, S. A. On the approximate solutions to a damped harmonic oscillator with higher-order nonlinearities and its application to plasma physics: Semi-analytical solution and moving boundary method. *Eur. Phys. J. Plus* **135**(10), 1–17 (2020).

[23] Wu, Y. & Liu, Y.-P. Residual calculation in He’s frequency–amplitude formulation. *J. Low Freq. Noise Vib. Active Control* **40**(2), 1040–1047 (2021).

[24] Ren, Z. F. & Hu, G. F. He’s frequency-amplitude formulation with average residuals for nonlinear oscillators. *J. Low Freq. Noise Vib. Active Control* **38**, 1050–1059 (2019).

[25] Qie, N., Houa, W.-F. & He, J.-H. The fastest insight into the large amplitude vibration of a string. *Rep. Mech. Eng.* **2**(1), 1–5 (2020).

[26] He, J.-H. Amplitude–frequency relationship for conservative nonlinear oscillators with odd nonlinearities. *Int. J. Appl. Comput. Math.* **3**, 1557–1560 (2017).

[27] Moatimid, G. M. & Mohamed, Y. M. Nonlinear electro-rheological instability of two moving cylindrical fluids: An innovative approach. *Phys. Fluids* **36**, 024110 (2024).

[28] Moatimid, G. M. & Mohamed, Y. M. A novel methodology in analyzing nonlinear stability of two electrified viscoelastic liquids. *Chin. J. Phys.* **89**, 679–706 (2024).

[29] Moatimid, G. M. & Mohamed, Y. M. Novel analytical perspectives on nonlinear instabilities of viscoelastic Bingham fluids in MHD flow fields. *Sci. Rep.* **14**, 28843 (2024). 39572603 10.1038/s41598-024-78848-8 PMC11582699

[30] Alanazy, A., Moatimid, G. M. & Mohamed, Y. M. Inspection of nonlinear instability of two cylindrical interfaces between viscoelastic magneto-rheological fluids. *Ain Shams Eng. J.* **16**(7), 103401 (2025).

[31] Munoz-Torres, J. R. et al. Biological properties and surgical applications of the human amniotic membrane. *Front. Bioeng. Biotechnol.* **10**, 1067480 (2023). 36698632 10.3389/fbioe.2022.1067480 PMC9868191

[32] Ronald, F. B. Petroleum Geology: An Introduction, New Mexico Bureau of Geology and Mineral Resources, Division of New Mexico Institute of Mining and Technology (2004).

[33] Ibrahim, W. & Hindebu, B. Magnetohydrodynamic (MHD) boundary layer flow of Eyring–Powell nanofluid past stretching cylinder with Cattaneo–Christov heat flux model. *Nonlinear Eng.* **8**, 303–317 (2019).

[34] Suriya, V. M. V. & Naveen, P. Exploration of flow irreversibility in electrically magnetized Powell–Eyring nanofluid along an inclined plate with viscous dissipation. *Cogent Eng.* **11**(1), 2386096 (2024).

[35] Challa, K. K. et al. Enhanced heat transfer and flow dynamics of Powell–Eyring nanofluid: Unsteady stretched surface and with Stefan blowing/suction. *Case Stud. Therm. Eng. Case Stud. Therm. Eng.* **65**, 105664 (2025).

[36] Kasali, K. B., Lawal, M. O., Mkhatshwa, M. P., Tijani, Y. O. & Otegbeye, O. Analysis of Eyring–Powell hybrid nanofluid flow with magnetic dipole over a stretching sheet. *Model. Earth Syst. Environ.* **11**, 396 (2025).

[37] Riaz, A., Shehzadi, M., Akram, S., Alhamzi, G. & Mahmoud, E. E. A peristaltic motion for pressure driven flow of Casson nanoliquid along with gyrotactic microorganisms in an entropic porous channel: A numerical study. *Mater. Today Commun.* **40**(1), 109971 (2024).

[38] Janaiah, Ch. & Reddy, B. S. Modelling of 3-D Casson-nano-Oldroyd-B fluid flow with Cattaneo–Christov double diffusion effects: applications in biomedical engineering. *Model. Earth Syst. Environ.* **11**(6), 412 (2025).

[39] Alhushaybari, A. et al. Thermal management analysis of thermal radiation effect on mixed convective Casson fluid inside wavy wall lid-driven cavity with diamond-shaped obstacles. *J. Radiat. Res. Appl. Sci.* **18**(3), 101808 (2025).

[40] Bahman, N., Fayyaz, A., Abbas, Z. & Rafiq, M. Y. Investigating entropy optimization in radiatively peristaltic transport of Casson fluid in the annular region of eccentric cylinders under lubrication hypothesis. *J. Radiat. Res. Appl. Sci.* **18**(3), 101715 (2025).

