# Supplementary material for: Advanced analysis of nonlinear stability of two horizontal interfaces separating three-stratified non-Newtonian liquids
Source: Sci Rep. 2025 Nov 18;15:40396. doi: 10.1038/s41598-025-24182-6 (PMC12627485; doi:10.1038/s41598-025-24182-6)
Supplement: Supplementary file 1 — Supplementary Material 1 [file 41598_2025_24182_MOESM1_ESM.docx]

**Appendix**

The constants outlined in Eqs. (5) and (8) may be given as:

$A_{1}(t) =\frac{- e^{-ak}\eta_{t}}{k\left(1+i\eta_{x}\right)},$ $A_{2}(t) =\frac{- e^{-ak}\xi_{t}\left(-i+\eta_{x}\right)+e^{3ak}\eta_{t}\left(-i+\xi_{x}\right)}{k\left(\left(1+i\eta_{x}\right)\left(i+\xi_{x}\right)+e^{4ak}\left(1-i\eta_{x}\right)\left(-i+\xi_{x}\right)\right)},$ $A_{3}(t) =\frac{e^{3ak}\xi_{t}\left(i+\eta_{x}\right)- e^{-ak}\eta_{t}\left(i+\xi_{x}\right)}{k\left(\left(1+i\eta_{x}\right)\left(i+\xi_{x}\right)+e^{4ak}\left(1-i\eta_{x}\right)\left(-i+\xi_{x}\right)\right)},$ $A_{4}(t) =\frac{ e^{ak}\xi_{t}}{k\left(1-i\xi_{x}\right)},$ $B_{1}(t) =\frac{E_{0}e^{ak}\Delta_{2}}{k\left(-i+\eta_{x}\right)\Delta_{1}},$ $B_{2}(t) =\frac{\left(\varepsilon_{1}-\varepsilon_{2}\right)e^{ak}E_{0}}{k\Delta_{1}}\left(\left(\varepsilon_{2}+\varepsilon_{3}\right)\eta_{x}\left(1+i \xi_{x}\right)e^{2ak}+ \left(\varepsilon_{2}-\varepsilon_{3}\right)\xi_{x}\left(1-i \eta_{x}\right)\right),$

$B_{3}(t) =\frac{\left(\varepsilon_{2}-\varepsilon_{3}\right)e^{ak}E_{0}}{k\Delta_{1}}\left(\left(\varepsilon_{1}-\varepsilon_{2}\right)\eta_{x}\left(1-i\xi_{x}\right)+e^{2ak}\left(\varepsilon_{1}+\varepsilon_{2}\right)\xi_{x}\left(1-i\eta_{x}\right)\right),$ $B_{4}(t) =\frac{E_{0}e^{ak}\Delta_{3}}{k\left(i+\xi_{x}\right)\Delta_{1}},$ $\Delta_{1}=e^{4ak}\left(\varepsilon_{1}+\varepsilon_{2}\right)\left(\varepsilon_{2}+\varepsilon_{3}\right)_{x}\left(i+\eta_{x}\right)\left(-i+\xi_{x}\right)+\left(\varepsilon_{1}-\varepsilon_{2}\right)\left(\varepsilon_{2}-\varepsilon_{3}\right)\left(-i+\eta_{x}\right)\left(i+\xi_{x}\right),$ $\begin{matrix} \Delta_{2}=-2ie^{2ak}\varepsilon_{2}\left(\varepsilon_{2}-\varepsilon_{3}\right)\xi_{x}\left(1+\eta_{x}^{2}\right)+e^{4ak}\left(\varepsilon_{1}-\varepsilon_{2}\right)\left(\varepsilon_{2}+\varepsilon_{3}\right)\eta_{x}\left(1-i\eta_{x}\right)\left(-i+\xi_{x}\right) \\ +\left(\varepsilon_{2}-\varepsilon_{1}\right)\left(\varepsilon_{2}-\varepsilon_{3}\right)\eta_{x}\left(1+i\eta_{x}\right)\left(i+\xi_{x}\right), \end{matrix}$

and

$$\begin{matrix} \Delta_{3}=\left(\varepsilon_{1}-\varepsilon_{2}\right)\left(\varepsilon_{2}-\varepsilon_{3}\right)\xi_{x}\left(1-i\xi_{x}\right)\left(-i+\eta_{x}\right)+e^{4ak}\left(\varepsilon_{1}+\varepsilon_{2}\right)\left(\varepsilon_{2}-\varepsilon_{3}\right)\xi_{x}\left(1+i\xi_{x}\right)\left(i+\eta_{x}\right) \\ +2ie^{2ak}\varepsilon_{2}\left(\varepsilon_{1}-\varepsilon_{2}\right)\eta_{x}\left(1+\xi_{x}^{2}\right), \end{matrix}$$

The factors appearing in Eqs. (21) and (22) may be expressed as:

$r_{1}=a_{1}/a_{0},$ $r_{2}=a_{2}/a_{0},$ $r_{3}=a_{3}/a_{0},$ $r_{4}=a_{4}/a_{0},$ $r_{5}=a_{5}/a_{0},$ $r_{6}=a_{6}/a_{0},$ $r_{7}=a_{7}/a_{0},$ $r_{8}=a_{8}/a_{0},$ $r_{9}=a_{9}/a_{0},$ $r_{10}=a_{10}/a_{0},$ $r_{11}=a_{11}/a_{0},$ $r_{12}=a_{12}/a_{0},$ $r_{13}=a_{13}/a_{0},$ $r_{14}=a_{14}/a_{0},$ $r_{15}=a_{15}/a_{0},$ $r_{16}=a_{16}/a_{0},$ $r_{17}=a_{17}/a_{0},$ $r_{18}=a_{18}/a_{0},$ $r_{19}=a_{19}/a_{0},$ $r_{20}=a_{20}/a_{0},$ $r_{21}=a_{21}/a_{0},$ $r_{22}=a_{22}/a_{0},$ $r_{23}=a_{23}/a_{0},$ $r_{24}=a_{24}/a_{0},$ $r_{25}=a_{25}/a_{0},$ and $r_{26}=a_{26}/a_{0}.$ As well,

$s_{1}=b_{1}/b_{0},$ $s_{2}=b_{2}/b_{0},$ $s_{3}=b_{3}/b_{0},$ $s_{4}=b_{4}/b_{0},$ $s_{5}=b_{5}/b_{0},$ $s_{6}=b_{6}/b_{0},$ $s_{7}=b_{7}/b_{0},$ $s_{8}=b_{8}/b_{0},$ $s_{9}=b_{9}/b_{0},$ $s_{10}=b_{10}/b_{0},$ $s_{11}=b_{11}/b_{0},$ $s_{12}=b_{12}/b_{0},$ $s_{13}=b_{13}/b_{0},$ $s_{14}=b_{14}/b_{0},$ $s_{15}=b_{15}/b_{0},$ $s_{16}=b_{16}/b_{0},$ $s_{17}=b_{17}/b_{0},$ $s_{18}=b_{18}/b_{0},$ $s_{19}=b_{19}/b_{0},$ $s_{20}=b_{20}/b_{0},$ $s_{21}=b_{21}/b_{0},$ $s_{22}=b_{22}/b_{0},$ $s_{23}=b_{23}/b_{0},$ $s_{24}=b_{24}/b_{0},$ $s_{25}=b_{25}/b_{0},$and $s_{26}=b_{26}/b_{0},$

where,$a_{0}=-m_{1}\left(\rho_{1}+m_{0}h_{2}\right),$ $a_{1}=m_{1}e^{ak}m_{0}h_{3},$ $a_{2}=-m_{1}m_{2}Oh\left(\mu_{1}+m_{0}h_{2}\right)-2kOh\left(\mu_{1}+\Lambda m_{0}\right)-2P_{e}kOh,$ $a_{3}=m_{0}m_{1}m_{2}Oh e^{ak}h_{3}+2m_{0}k \Lambda Oh e^{2ak},$ $a_{4}=B_{d}\left(\rho_{1}-1\right)-k^{2},+km_{3} \left(\varepsilon_{1}^{2}-1\right)E_{0}^{2}\left(h_{1}+\varepsilon_{3}h_{4}\right),$ $a_{5}=2e^{2ak}km_{3} \left(1-\varepsilon_{1}\right) \left(\varepsilon_{1}-1\right)E_{0}^{2},$ $a_{6}=-\rho_{1}+h_{2}m_{0}Q_{1},$ $a_{7}=m_{0}Q_{1}e^{ak}h_{3},$ $a_{8}=-m_{0}e^{ak}\left(h_{5}+Q_{1}h_{3}\right),$ $a_{9}=-m_{0}\left(h_{6}+Q_{1}h_{2}\right),$ $a_{10}=-m_{0} Oh m_{2}\left(h_{6}+Q_{1}h_{2}\right)-2k^{2}m_{0} Oh \Lambda\left(1+Q_{1}\right),$ $a_{11}=-Oh m_{0} m_{2}e^{ak} \left(h_{5}+Q_{1}h_{3}\right)-2k^{2}m_{0} Oh \Lambda e^{2ak}\left(1+Q_{1}\right),$ $a_{12}=2k^{2}P_{e}Oh+m_{0} Oh m_{2} \left(Q_{1}h_{2}-\mu_{1}q_{1}\right)+2m_{0}k^{2}Oh\left(\mu_{1}q_{1}+\Lambda\left(2+Q_{1}\right)\right),$ $a_{13}=m_{0} m_{2} Oh e^{ak}Q_{1}h_{3}+2k^{2}m_{0}Oh\Lambda e^{2ak}Q_{1},$ $a_{14}=a_{140}+a_{141},$ $a_{140}=-0.5m_{4}k^{2}E_{0}^{2}\left(2\varepsilon_{1}s_{0}^{2}-2s_{0}\left(-1+\varepsilon_{1}\right)\left(-\left(2+R_{1}\right)\left(1+\varepsilon_{1}\right)\left(-1+\varepsilon_{3}\right)+e^{4ak}\left(-2+R_{1}\left(1+\varepsilon_{1}\right)\right)\left(1+\varepsilon_{3}\right)\right)\right),$

$a_{141}=-0.5m_{4}k^{2}E_{0}^{2}\left(-1+\varepsilon_{1}\right)^{2}\left(2\left(-1+\varepsilon_{1}\right)\left(-1+\varepsilon_{3}\right)^{2}+2e^{8ak}\left(-1+\varepsilon_{1}\right)\left(1+\varepsilon_{3}\right)^{2}-4e^{4ak}\varepsilon_{1}\left(-1+\varepsilon_{3}^{2}\right)\right),$ $a_{15}=2k^{2}e^{2ak}m_{4} E_{0}^{2}\left(-1+\varepsilon_{3}\right)\left(-\left(1+\varepsilon_{1}\right)R_{1}s_{0}+e^{2ak}\left(-1+\varepsilon_{3}\right)\left(-1+\varepsilon_{1}\right)^{2}\right),$ $a_{16}=-m_{0}h_{5}h_{3}^{2},$ $a_{17}=a_{170}+a_{171},$

$$\begin{matrix} a_{170}=-1.5k^{4}+k^{3}m_{4} E_{0}^{2}\left(-1+\varepsilon_{1}\right)^{2}\left(2\left(1+R_{1}\right)\left(-1+\varepsilon_{1}\right)\left(-1+\varepsilon_{3}\right)^{2}+2e^{8ak}\left(-1+R_{1}\right)\left(-1+\varepsilon_{1}\right)\left(1+\varepsilon_{3}\right)^{2}\right)-4k^{3}e^{8ak} \\ m_{4} R_{1}\varepsilon_{1}E_{0}^{2}\left(-1+\varepsilon_{1}\right)^{2}\left(-1+\varepsilon_{3}^{2}\right), \end{matrix}$$

$a_{171}=k^{3}m_{4} E_{0}^{2}\left(-1+\varepsilon_{1}\right)s_{0}\left(-2\varepsilon_{1}\left(-1+\varepsilon_{3}\right)-2R_{1}\left(\left(1+\varepsilon_{1}\right)\left(-1+\varepsilon_{3}\right)+e^{4ak}\left(1+\varepsilon_{3}\right)\right)+2R_{1}^{2}\left(-1+\varepsilon_{1}\right)\left(1-\varepsilon_{3}+e^{4ak}\left(1+\varepsilon_{3}\right)\right)\right),$ $a_{18}=-2k^{3}R_{1}\varepsilon_{1}e^{2ak}m_{4} E_{0}^{2}\left(-1+\varepsilon_{3}\right)\left(\left(1+\varepsilon_{1}\right)R_{1}s_{0}-2e^{2ak}\left(-1+\varepsilon_{3}\right)\left(-1+\varepsilon_{1}\right)^{2}\right),$

$a_{19}=-km_{0}\left(Q_{1}^{2}h_{2}+q_{1}\rho_{1}\right),$ $a_{20}=ke^{ak}m_{0}Q_{1}^{2}h_{3},$ $a_{21}=-km_{0}Q_{1}\left(h_{6}+h_{2}Q_{1}\right),$ $a_{22}=-ke^{2ak}m_{0}Q_{1}\left(h_{5}+h_{3}Q_{1}\right),$

$$a_{23}=-m_{0}Oh km_{2}\left(q_{1}\mu_{1}+Q_{1}^{2}h_{2}\right)-2P_{e}Ohk^{3}-2m_{0}k^{3}Oh\left(\left(q_{1}\mu_{1}+h_{2}\right)+\Lambda\left(1+Q_{1}\right)^{2}\right),$$

$a_{24}=Oh e^{ak}m_{0}km_{2}Q_{1}^{2}h_{3}+2k^{3}m_{0}Oh\Lambda Q_{1}^{2}e^{2ak},$ $a_{25}=-Oh m_{0} km_{2}Q_{1}\left(h_{6}+Q_{1}h_{2}\right)-2k^{3}m_{0}Q_{1}Oh\Lambda\left(1+Q_{1}\right),$ $a_{26}=Oh m_{0} km_{2}Q_{1}e^{ak}\left(h_{5}+Q_{1}h_{3}\right)+2k^{3}m_{0}Oh\left(e^{ak}h_{3}+Q_{1}\Lambda e^{2ak}\left(-1+Q_{1}+Q_{1}^{2}\right)\right),$ $Q_{1}=m_{0}h_{1},$ $h_{1}=1+e^{4ak},$ $h_{2}=1+e^{3ak},$ $h_{3}=1+e^{ak},$ $h_{4}=1-e^{4ak},$ $h_{5}=1-e^{ak},$ $h_{6}=1-e^{3ak},$ $q_{1}=-1+e^{4ak},$ $q_{2}=1-e^{3ak},$ $q_{3}=1-e^{ak},$ $R_{1}=m_{3}h_{2},$ $m_{0}=q_{1}^{-1},$ $m_{1}=k^{-1},$ $m_{2}=D_{n}^{-1},$ $m_{3}=s_{0}^{-1},$ $m_{4}=s_{0}^{-2},$and$s_{0}=h_{1}\left(\varepsilon_{1}+\varepsilon_{3}\right)+q_{1}\left(1+\varepsilon_{1}\varepsilon_{3}\right).$ Moreover,

$b_{0}=-m_{1}\left(\rho_{3}+e^{ak}h_{2}m_{0}\right),$ $b_{1}=m_{0}m_{1}e^{2ak}h_{3},$ $b_{2}=-2kOhP_{e}-m_{1}m_{2}Oh\left(\mu_{3}+e^{ak}h_{3}m_{0}\right)-2kOh\left(\mu_{3}+e^{4ak}\Lambda m_{0}\right)$ $b_{3}=Oh e^{2ak}h_{3}m_{0} m_{1} m_{2}+2m_{0}k\Lambda Ohe^{2ak},$ $b_{4}=B_{d}\left(-\rho_{3}+1\right)-k^{2}-m_{3}kE_{0}^{2}\left(h_{1}-\varepsilon_{1}h_{4}\right)\left(1-\varepsilon_{3}^{2}\right),$ $b_{5}=-2m_{3}e^{2ak}kE_{0}^{2}\left(\varepsilon_{1}-1\right)\left(\varepsilon_{3}+1\right),$ $b_{6}=\rho_{3}-m_{0}Q_{1}e^{ak}h_{2},$ $b_{7}=-m_{0}Q_{1}e^{2ak}h_{3},$ $b_{8}=m_{0}e^{ak}\left(h_{6}+h_{2}Q_{1}\right),$ $b_{9}=m_{0}e^{2ak}\left(h_{5}+h_{3}Q_{1}\right),$

$b_{10}=m_{0} m_{2} Ohe^{ak}\left(h_{6}+h_{2}Q_{1}\right)+2m_{0}k^{2}Oh\Lambda e^{4ak}\left(-1+Q_{1}\right),$ $b_{11}=m_{0} m_{2} Ohe^{2ak}\left(h_{5}+h_{3}Q_{1}\right)+2m_{0}k^{2}Oh\Lambda e^{2ak}\left(-1+Q_{1}\right),$ $b_{12}=-2k^{2}P_{e}Oh-m_{0} Oh m_{2} \left(Q_{1}h_{2}e^{ak}-\mu_{3}q_{1}\right)-2m_{0}k^{2}Oh\left(\mu_{3}q_{1}+\Lambda e^{4ak}\left(-2+Q_{1}\right)\right),$ $b_{13}=-m_{0} m_{2} Oh e^{2ak}Q_{1}h_{3}-2k^{2}m_{0}Oh\Lambda e^{2ak}Q_{1},$ $b_{14}=b_{140}+b_{141},$ $b_{140}=-m_{4}k^{2}E_{0}^{2}\left(s_{0}^{2}+s_{0}\left(1-\varepsilon_{3}\right)\left(-\left(2+R_{1}\right)\left(-1+\varepsilon_{1}\right)\left(1+\varepsilon_{3}\right)+e^{4ak}\left(-2+R_{1}\left(1+\varepsilon_{3}\right)\right)\left(1+\varepsilon_{1}\right)\right)\right),$

$a_{141}=-m_{4}k^{2}E_{0}^{2}\left(-1+\varepsilon_{3}\right)^{2}\left(-\left(-1+\varepsilon_{3}\right)\left(-1+\varepsilon_{1}\right)^{2}-e^{8ak}\left(-1+\varepsilon_{3}\right)\left(1+\varepsilon_{1}\right)^{2}+2e^{4ak}\varepsilon_{3}\left(-1+\varepsilon_{1}^{2}\right)\right),$ $b_{15}=-2k^{2}e^{2ak}m_{4} E_{0}^{2}\left(-1+\varepsilon_{1}\right)\left(-\left(1+\varepsilon_{3}\right)R_{1}s_{0}+e^{2ak}\left(-1+\varepsilon_{1}\right)\left(-1+\varepsilon_{3}\right)^{2}\right),$ $b_{16}=m_{0}e^{ak}h_{5}h_{2}^{2},$ $b_{17}=b_{170}+b_{171},$

$$\begin{matrix} b_{170}=-1.5k^{4}-2k^{3}m_{4} E_{0}^{2}\left(1-\varepsilon_{3}\right)^{2}\left(-\left(1+R_{1}\right)\left(-1+\varepsilon_{3}\right)\left(-1+\varepsilon_{1}\right)^{2}-e^{8ak}\left(-1+R_{1}\right)\left(-1+\varepsilon_{3}\right)\left(1+\varepsilon_{1}\right)^{2}\right)+2k^{3}e^{4ak} \\ m_{4} R_{1}\varepsilon_{3}E_{0}^{2}\left(-1+\varepsilon_{1}^{2}\right)\left(1-\varepsilon_{3}\right)^{2}, \end{matrix}$$

$b_{171}=-k^{3}m_{4} E_{0}^{2}\left(1-\varepsilon_{3}\right)s_{0}\left(e^{4ak}\left(2\left(1-R_{1}-\varepsilon_{3}\right)+\left(1+\varepsilon_{3}\right)R_{1}^{2}\right)\left(1+\varepsilon_{1}\right)-\left(2\left(1+R_{1}\left(1+\varepsilon_{3}\right)\right)+\left(1+\varepsilon_{3}\right)R_{1}^{2}\right)\left(-1+\varepsilon_{1}\right)\right),$ $b_{18}=-2k^{3}R_{1}e^{2ak}m_{4} E_{0}^{2}\left(1-\varepsilon_{1}\right)\left(\left(1+\varepsilon_{3}\right)R_{1}s_{0}-2e^{2ak}\left(\varepsilon_{1}-1\right)\left(1-\varepsilon_{3}\right)^{2}\right),$

$b_{19}=-km_{0}\left(Q_{1}^{2}h_{2}e^{ak}+q_{1}\rho_{3}\right),$ $b_{20}=ke^{2ak}m_{0}Q_{1}^{2}h_{3},$ $b_{21}=-ke^{ak}m_{0}Q_{1}\left(h_{6}+h_{2}Q_{1}\right),$ $b_{22}=ke^{2ak}m_{0}Q_{1}\left(h_{5}+h_{3}Q_{1}\right),$

$$b_{23}=-m_{0}Oh km_{2}\left(q_{1}\mu_{3}+e^{ak}Q_{1}^{2}h_{2}\right)-2P_{e}Ohk^{3}-2m_{0}k^{3}Oh\left(q_{1}\mu_{3}+\Lambda e^{ak}\left(1+e^{3ak}\left(2\left(1-Q_{1}\right)+Q_{1}^{2}\right)\right)\right),$$

$b_{24}=Oh e^{2ak}m_{0}km_{2}Q_{1}^{2}h_{3}+2k^{3}m_{0}Oh\Lambda Q_{1}^{2}e^{2ak},$ $b_{25}=-Oh m_{0}e^{ak} km_{2}Q_{1}\left(h_{6}+Q_{1}h_{2}\right)-2k^{3}e^{4ak}m_{0}Q_{1}Oh\Lambda\left(-1+Q_{1}\right),$and $b_{26}=Oh m_{0} km_{2}Q_{1}e^{2ak}\left(h_{5}+Q_{1}h_{3}\right)+2k^{3}m_{0}Oh\Lambda\left(e^{ak}+Q_{1}\left(-1+Q_{1}\right)\right).$
